# A general-purpose Nanohybrid fabricated by Polymeric Au(I)-peptide precursor to wake the function of Peptide Therapeutics

**DOI:** 10.7150/thno.47243

**Published:** 2020-07-09

**Authors:** Jin Yan, Fanpu Ji, Siqi Yan, Weiming You, Fang Ma, Fanni Li, Yinong Huang, Wenjia Liu, Wangxiao He

**Affiliations:** 1 National & Local Joint Engineering Research Center of Biodiagnosis and Biotherapy, The Second Affiliated Hospital of Xi'an Jiaotong University, Xi'an, 710004, PR. China.; 2 Department of Tumor and Immunology in precision medical institute, Western China Science and Technology Innovation Port, Xi'an, 710004, PR. China.; 3 Department of Infectious Diseases, The Second Affiliated Hospital of Xi'an Jiaotong University, Xi'an, 710004, PR. China.; 4 Key Laboratory of Environment and Genes Related to Diseases, Xi'an Jiaotong University, Ministry of Education of China, Xi'an, China; 5 Department of Talent Highland, The First Affiliated Hospital of Xi'an Jiaotong University, Xi'an 710061, PR. China.; 6 Shaanxi Institute of Pediatric Diseases, Xi'an Children's Hospital, Xi'an, Shaanxi 710003, PR. China.; 7 The Second Affiliated Hospital of Xi'an Jiaotong University, Xi'an, 710004, PR. China.

**Keywords:** peptide, nanoparticle, anti-cancer, peptide therapeutics, protein-protein interactions

## Abstract

Peptide-derived nanocomposites have been exhibiting fascinating biological advantages, including but not limited to excellent biocompatibility, biological degradation, high targetability and subsequent potent therapeutic efficacy. While some successes have been achieved in the nanoengineering of peptide-based architectures with defined dimensions and medical functions, enormous challenges remain about clinical nano-pharmaceutics of peptides, especially those modulating intracellular protein-protein interactions (PPIs).

**Methods:** We developed a general method to translate intracellular-PPI-targeted peptides into a bioavailable peptide-auric **s**pheroidal **n**ano**h**ybrid (SNH), for which polymeric peptide-Auric precursors [Au^1+^-S-peptide]_n_ are *in-situ* reduced on the surface of gold nanoseeds *via* a simple and mild reaction. As proofs of concept, three cytomembrane-impenetrable peptides with different physicochemical properties were successfully engineered into stable and tumor-specific SNH respectively.

**Results:** To highlight the advantage of SNH, PMI, a hydrophobic and enzyme-intolerant peptide capable of p53 restoration, was selected to challenge the power of SNH in a colon tumor xenografts model. PMI-Au SNH *in vivo* suppressed tumor growth potently after three administrations: intravenous injection, intraperitoneal injection and gastric perfusion, and maintained a favorable therapeutic safety.

**Conclusion:** This therapeutically feasible strategy of peptide nanoengineering will allow us to fabricate a series of nanomedicines to modulate carcinogenic PPIs that hide and multiply inside cells, and in all likelihood reinvigorate the development of peptide drug against wide varieties of human diseases.

## Introduction

Intracellular Protein-Protein Interactions (PPIs) play a pivotal part in all biological systems and are often dysregulated in diseases, representing an important, and yet largely unexploited, class of therapeutic targets 1. It has been estimated that human physiological activities involve over four hundred thousand intracellular PPIs, offering a plenty of opportunities for pharmacological interventions against a wide variety of diseases 2, 3. Nevertheless, the overwhelming classes of PPI are unamenable to small-molecule inhibition, which are mainly attributed to the generally flat and large interfaces of PPI (~800-2000 A^2^) in sharp contrast to the deep grooves that befit the small-molecular affinity (~300-500 A^2^) 3, 4. Fortunately, with the emergence of peptide therapeutics, hopeful signs are emerging. Benefitting from larger surfaces, peptides can closely mimic the topological features of protein, and as a result, peptides have emerged as the likeliest candidate for PPIs modulators 2.

In reality, however, peptide therapeutics, particularly those targeting intracellular PPIs, always suffer from two inherent pitfalls: poor proteolytic stability and low cytomembrane permeability 2, 5. To alleviate these pharmacological hurdles, a fast-growing number of choreographed modifications for proteolytic resistance and well-designed vehicles for targeted delivery have emerged in ways of clinical peptide translations 2, 5-7. Notwithstanding some successes in optimizing therapeutic peptides by these two methods, it is still challenging to convert intracellular PPIs into clinical trials, and no drug, as yet, has been approved for clinical application in this target class to our knowledge 8-10. Therefore, a lot of efforts remain to be done as regards bridging the gap between peptide discovery and clinical application.

In recent years, nanotechnology has afforded a bottom-up approach to convert peptides covalently or noncovalently into stable architectures with proteolytic resistance and cytomembrane penetrability 11-14. Some peptide-derived nanomedicines including liposome/macromolecule-derived peptide nanomicelles 15, peptide-coated nanoparticles 16-18 and peptide-based self-assembled nano-architectures 19-21, have exhibited attractive biological advantages including prolonged circulation time, enhanced disease specificity, intensified proteolytic stability and subsequent optimized therapeutic efficacy 11, 12. Among them, an increasing number of gold nanoparticles (AuNP)-conjugated peptide have been developed and applied for clinical trials in because of its intrinsic advantage including essential inertion, low-toxicity and economic costs 22-25. Up to now, AuNPs have provided non-toxic carriers for drugs and biomolecules delivery applications 26-28. Yet the complex chemical properties (hydrophobicity, charge and redox) of peptide are always adverse to the steady state of colloidal AuNP after conjugation, resulting in the subsequent aggregation or even precipitation under the physiological condition of the elevated ionic concentration 29, 30. Moreover, the weakened colloidal stability often accompanies premature release of therapeutic peptide and enhanced reticuloendothelial system uptakes, and ultimately leads to off-target toxicity and therapeutics failing 26, 31.

To address them, we turn our gaze to a *de novo* synthesis of nanoparticle by Au(I) thiolate precursors 32, 33. By this approach, previous reports successfully fabricated size-tuned gold nanoparticles *via* reducing Au(I)-glutathione precursors 32, 34. But that reaction, in which thiol peptide strains the conversion of the ionic gold precursor into metallic gold nuclei, has to be driven by strong reducing agent, such as sodium borohydride (NaBH_4_) 33. As a result, peptides are likely to be destroyed in such harsh reaction condition, and thus, there is a critical need for a mild alternative.

**Figure 1:**
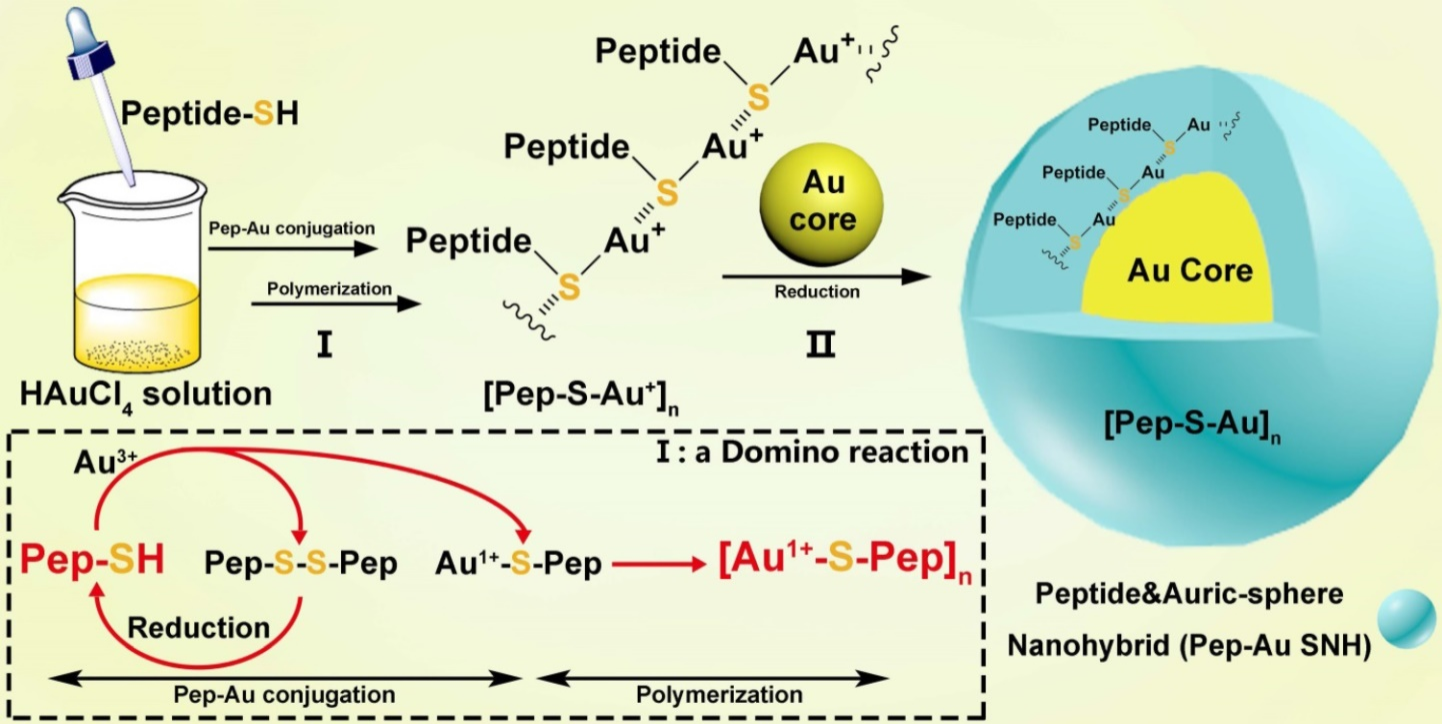
** Schematic depiction for peptide-Au SNH synthesis.** The chemistry for SNH formation consists of two reaction: I) a domino reaction in copolymerization between thiol peptide and Au ions to synthesize Au-peptide precursor and, II) reducing polymeric precursor at the surface of prefabricated ultra-small gold seed.

For these reasons, we herein developed a general method to convert therapeutic peptides into a stable and bioavailable auric **s**phere **n**ano**h**ybrid (Au SNH) by a mild and simple chemistry route. In this case, peptide-auric precursors are reduced by hydroxyethyl piperazine ethylsulfonic acid (HEPES) at the surface of prefabricated ultra-small gold seed (Figure 1). Of note, the using of prefabricated gold seed as nuclei detoured the harsh reaction condition for the conversion of the ionic gold precursor into gold nuclei, guaranteeing the biological activity of peptides. In the proof-of-concept study, three cytomembrane-impenetrable anti-cancer peptides were copolymerized with chloroauric acid to form peptide-auric **s**pheroidal **n**ano**h**ybrid (SNH): 1) a 12-mer hydrophobic and enzyme-intolerant p53 activator, termed PMI, 2) a 20-mer hydrophilic Wnt inhibitor, termed BBI, and 3) a 12-mer hydrophobic and dextrorotary (proteolytic-resistive) p53 activator, termed DPA. As expected, SNH rescued the biofunction of three peptides that, on their own, failed to kill cancer cells. To highlight the advantage of SNH, the most hydrophobic and fragile one in the three peptides, PMI, was selected to challenge the power of SNH in a colon tumor xenografts model through three administrations: intravenous injection, intraperitoneal injection and gastric perfusion. This work amply confirmed the design of peptide-auric SNH as a general and viable strategy of nano-pharmaceutic to concert therapeutic peptides into potential drugs.

## Result

### Fabrication of peptide-Au SNH

Broadly, the chemistry for SNH formation consists of two reaction: Ⅰ) a domino reaction in copolymerization between thiol peptide and Au ions to synthesize Au-peptide precursor and II) reducing polymeric precursor at the surface of prefabricated ultra-small gold seed (Figure 1). For the embodiment of the chemistry, PMI-SH (seq.: TSFAEYWALLSPC), a cysteine-modified dodecameric peptide antagonist of MDM2 for p53 restoration, was firstly exploited to synthesize PMI-Au SNH.

**Figure 2:**
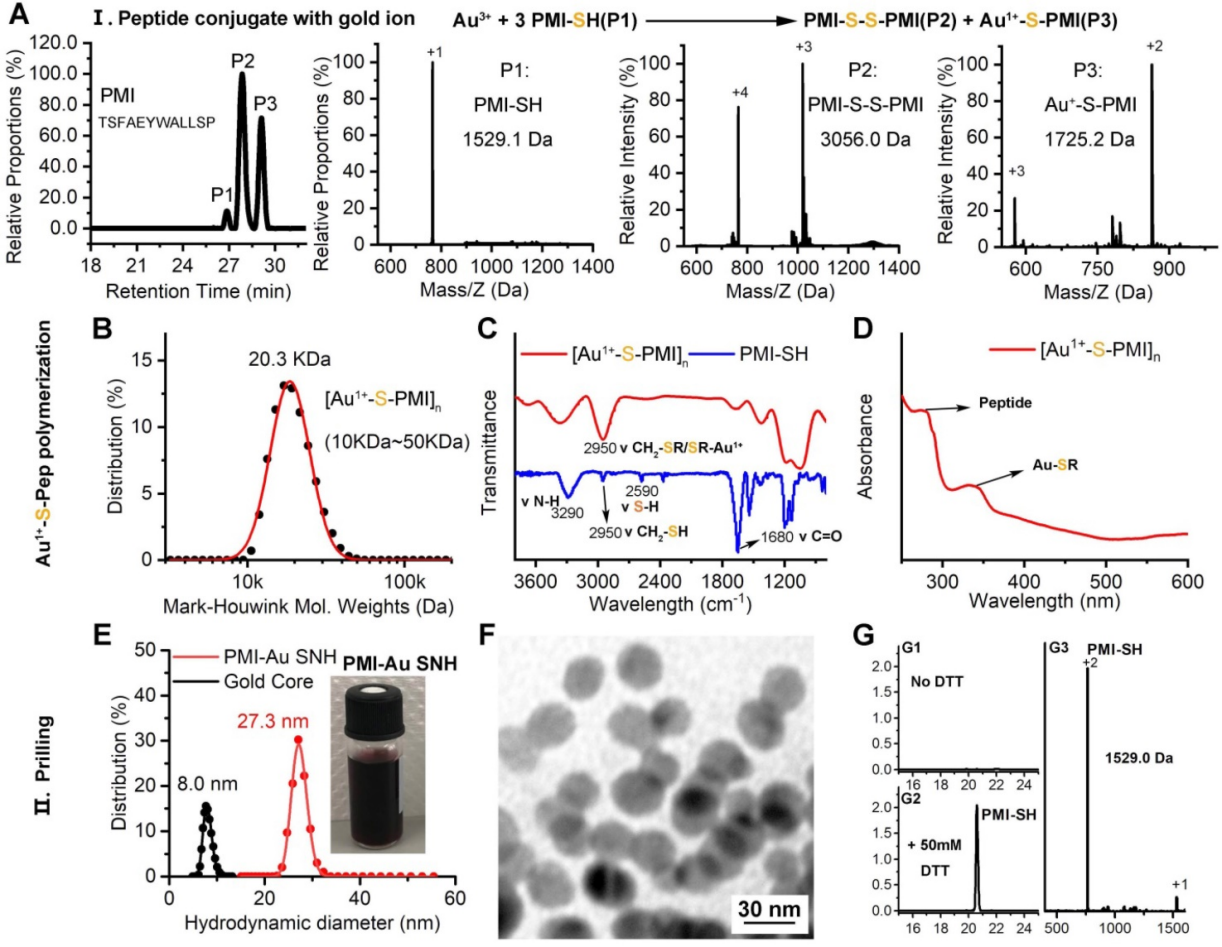
** Preparation and Characterization of PMI-Au SNH.** (**A**) The High-Performance Liquid Chromatography (HPLC) analysis and ESI-MASS results of the three peaks, suggesting that Au^+^-S-peptide complex was successfully synthesized. (**B**) Molecular weight distribution of the [Au^+^-S-PMI]n polymers measured by the Mark-Houwink-Sakurada method, which uses empirical constants to calculate the molecular weight from the diffusion coefficient determined from the autocorrelation function of the scattered light (DLS). (**C**) FT-IR spectra of [Au^+^-S-PMI]n and PMI-SH. The characteristic peak of free thiol at 2590 cm^-1^ in PMI-SH disappeared, and a new peak at 2950 cm^-1^ appeared in the spectroscopy of [Au^+^-S-PMI]n. These results demonstrated the chemical bonds of S-Au were formed. (**D**) UV-Vis absorption spectra of [Au^+^-S-PMI]n. The distinct absorption peaks at 330 nm in the UV-Vis region is the absorption peaks for the Au-S-peptide species. (**E**) Hydrodynamic diameter distributions of PMI-Au SNH and Gold Core, and the solution photo of PMI-Au SNH. (**F**) transmission electron micrograph images (TEM) of PMI-Au SNH. (**G**) HPLC analysis of the residual PMI-SH in the liquid supernatant after the PMI-Au SNH synthesis and centrifugation (G1). G2 is the HPLC analysis of PMI-Au SNH-redissolved solution including 50 mM dithiothreitol (DTT), and G3 is the ESI-MASS result of the peak in G2.

In the domino reaction Ⅰ (Figure 1), the [Au^1+^-S-pep] complex was produced by the coordination between the ionized HAuCl_4_ (Au^3+^) in HEPES buffer (pH 7.4) and the thiol group in the thiol-peptide (pep-SH) 32. The formation of [Au^1+^-S-PMI] was substantiated using a liquid chromatographic method with mass spectrometric detection and identification (LC-MS), by which the molecular mass of the product in peak P3 was 196.1 Da higher than that of the substrate (PMI-SH) in peak P1, in agreement with the molecular weight of the [Au^1+^-S-PMI] monomer (Figure 2A). Besides, the peak 2 proved the formation of the oxidized dimer of PMI-SH, indicative of the reaction equation for [Au^1+^-S-PMI] formation in Figure 2A. Subsequently, the polymerization of [Au^1+^-S-Pep] will spontaneously start in this chemical environment 35, as a result that the clear and transparent colorless solution changed to milky. When the turbidity was not aggravating, hardly any intermediate [Au^1+^-S-PMI] and PMI-S-S-PMI and the substrate PMI-SH can be detected (Figs. S1A-B), indicating the completeness of this domino reaction. At this point, the polymeric [Au^1+^-S-PMI] can be detected and proved by its molecular weight (Figure 2B), Fourier-transform infrared spectroscopy (FT-IR, Figure 2C) and UV-vis spectroscopy (Figure 2D). In line with the reaction mechanism previously reported that Au^1+^ ions are bridged with the mercapto group of pep-SH *via* a 2-coordinate chemical link (Figure 1) 32, a significantly increased absorption peak of Au^+^-SR vibration in FT-IR at 2950 cm^-1^ (Figure 2C) and a characteristic peak of Au^+^-SR absorption in UV-vis at 330 nm (Figure 2D) can be found in the sample of [Au^1+^-S-PMI]_n_ 35, 36.

Next, as for reaction II, isopycnic 50 mM HEPES solution (pH 7.4) containing 1mM seed gold nanoparticles (Gold Core) were added into the precursor mixture (Figure 1A). Owing to the aurophilic interaction and super van der Waals bonding^37^, ^38^, [Au^1+^-S-PMI]_n_ can be reduced on the surface and conjugated to Gold Core, resulting in the maturation into a spherical nanohybrid. After half-an-hour stirring, the resultant reaction mixture converted back to be clear and transparent (Figure 2E), and the purple solution portended the successful synthesis of PMI-Au SNH. The construction was first suggested by UV-vis absorption spectrum (Figure S1C), which shows a peak at ~280 nm and a surface plasma resonance at ~560nm. Of note, no fluorescence emission was found at λex = 280 nm (Figure S1D), which was presumably because of the fluorescence quenching ability of Au-core 39, 40. As shown in the dynamic light scattering (DLS) measurement in Figure 1E, the hydrodynamic diameter of PMI-Au SNH was 27.3±5.0 nm with the homogenous size distribution proved by the unimodal distribution and polydispersity index of 0.19, which showed an obvious increase in comparation to Au-Core (Figure 1E and S1E). Moreover, in Transmission Electron Microscope (TEM) images, PMI-Au SNH presented the monodispersed spherical structure ranged from 22 to 30 nm (Figure 1F), and the high-resolution TEM image showed the core-shell structure (Figure S1F). Moreover, the EDS analysis showed that the observed PMI-Au SNH was comprised of Au, N, O and S (Figure S1G) in line with the constitute of peptide and Au.

To determine the PMI-SH loading efficiency, we centrifugally removed the nanoparticle and quantified the residual PMI-SH in supernatant. To verify that all of the nanoparticle in supernatant was removed by 10000 g centrifugation, a time-dependent absorbance detection of supernatant at 530nm was performed. As shown in Figure S1H, nearly all of the nanoparticle had deposited after 30-min centrifugation. Surprisingly, no PMI-SH was found, indicating the approximate 100% loading efficiency (Figure 2G1). For further quantification, the centrifugated deposit was freeze-dried and weighted, following a resolution in 50mM dithiothreitol (DTT) to break the Au-S bond in PMI-Au SNH. In subsequent quantification by HPLC (Figure 2G2) and identification (Figure 2G3), the recovery of PMI-SH reached up to 92±3%, and the loading of PMI-SH in PMI-Au SNH was 72±8% (w_PMI-SH_/w_PMI-Au SNH_). Of note, the loading of therapeutic peptide in SNH much higher than that of previous AuNP-based nanomedicines 41, 42, were mainly caused by the skillful use of peptide cargo as part of the building blocks. Of note, the size of peptide-Au SNH nanoparticles can be regulated by different pH during synthesize, which can be proved by the DLS results (Figure S1I).

### The colloidal peptide-auric SNH is stable and proteolytically resistant

**Figure 3:**
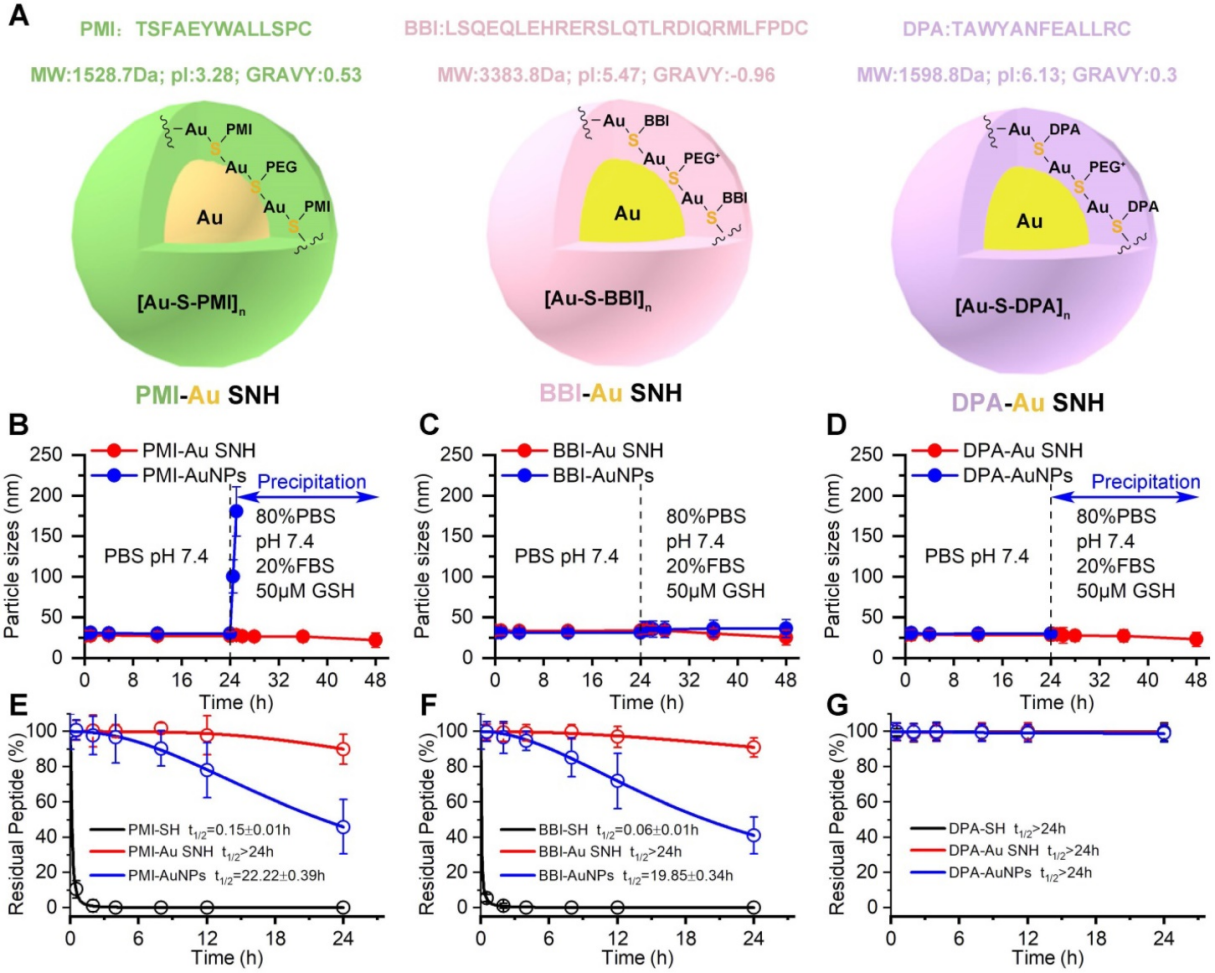
** The colloidal peptide-auric SNH is stable and proteolytically resistant.** (**A**) The physicochemical properties of the three peptide and the schematic diagram of three peptide-Auric SNH. (**B-D**) The dimensional change of Pep-AuNPs and Pep-Au SNH with time in PBS (pH7.4) or PBS including 20% FBS and 50 µM GSH to simulate extracellular physiological environment. (**E-G**) Proteolysis resistance of Pep-SH, Pep-AuNPs and Pep-Au SNH under PBS containing 10 mM oxidized glutathione, 10% serum, and 0.5 mg/ml chymotrypsin.

With the same method, BBI-Au SNH (Figure S2) and DPA-Au (Figure S3) SNH were also successfully synthesized (Figure 3A). For comparative studying the colloidal stability and proteolytic resistance of peptide-auric SNH, we prepared PMI-AuNPs, BBI-AuNPs and DPA-AuNPs by the conventional approach where peptide-Cys covalently conjugated to the surface of AuNP with ~30 nm diameter 18, 41, 42. We firstly comparatively examined the colloidal stability between Pep-AuNPs and Pep-Au SNH by suspending them, respectively, in PBS buffer at pH 7.4, and measuring time-dependent changes of hydrodynamic diameter *via* DLS. As shown in Figure 3B-D, all nanoparticles maintained almost unchanged in hydrodynamic diameter over 24 hours. The addition of 20% FBS and 50 μM GSH broke the stabilization, PMI-AuNPs (Figure 3B) and DPA-AuNPs (Figure 3D) precipitously precipitated out of buffer, while others remained monodispersed (Figure 3B-D). The reason, presumably, is that the conjugation with hydrophobic peptide can harmed the electric double layer of the colloidal gold 43, resulting the sub-stability under conditions of the elevated ionic concentration. In stark contrast, our *de novo* approach of SNH synthesis shuns the interaction between the peptide and formed colloidal particle, thereby protecting the integrity of electric double layer. For further verification, optical photographs and TEM images of three Au SNH were taken after 12 h PBS incubation or 12 h FBS/GSH (in PBS) incubation. As shown in Figure S4, three Au SNH maintain monodisperse. In general, these results suggested that the conventional approach to the synthesis of peptide-Au nanoparticles is just compatible with hydrophilic peptides, like BBI, and our *de novo* approach of SNH synthesis may be appropriate for all hydrophilic or hydrophobic peptides.

Linear peptides are conformationally disordered in aqueous solutions under their own steam and, consequently, tendentious to proteolysis. Conjugation of peptides with nanoparticles would improve steric hindrance against peptidase, resulting in the resistance to proteolysis 5, 44. We comparatively quantified the susceptibility of Pep-AuNPs and Pep-Au SNH to chymotrypsin using HPLC (Figure 3E-G). PMI-Au SNH and BBI-Au SNH were significantly more resistant to chymotrypsin-mediated proteolysis than their corresponding conventional AuNPs, PMI-AuNPs and BBI-AuNPs (Figure 3E and F), presumably because AuNPs exposed all its cargo on the surface in contrast to the effective protection in SNH. Notably, both of DPA-Au SNH and DPA-AuNPs showed excellent resistibility against chymotrypsin due to the intrinsic proteolytic resistance of dextrorotary peptide 20, 45.

### Peptide-auric SNH can traverse the cell membrane and GSH-triggered release cargo

**Figure 4:**
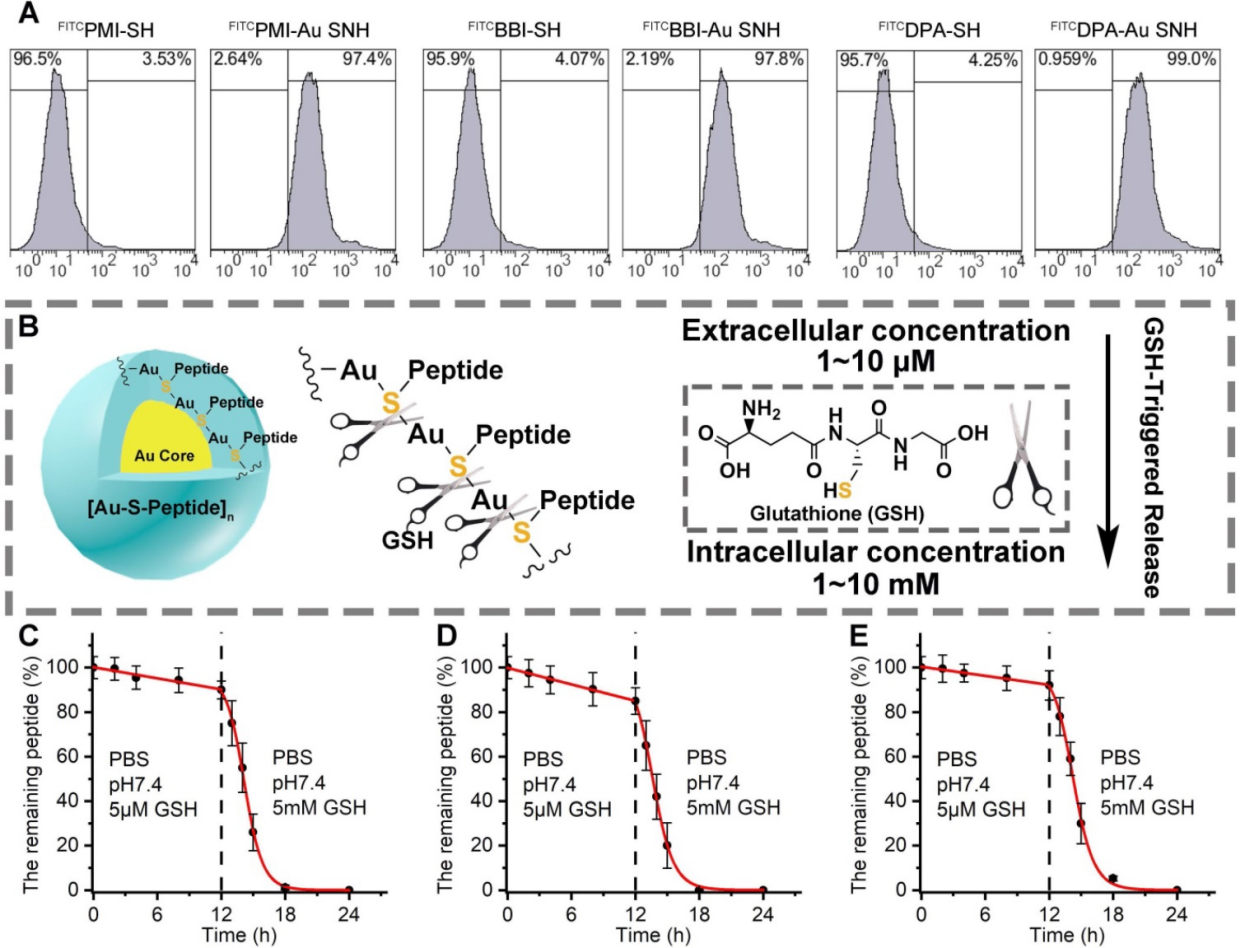
** Peptide-auric SNH can traverse the cell membrane and GSH-triggered release cargo.** (**A**) flow cytometry analysis of cell uptakes of 1 µM ^FITC^PMI-Au SNH, ^FITC^PMI,^ FITC^BBI-Au SNH, ^FITC^BBI, ^FITC^DPA-Au SNH and ^DPA^PMI into HCT116 cancer cells after 6h incubations. (**B**) schematic diagram of stimuli-responsive release of Pep-Au SNP triggered by intracellular glutathione (GSH). (**C-E**) PMI release from PMI-Au SNH (C), BBI release from BBI-Au SNH (D) and DPA release from DPA-Au SNH (D) under two different conditions that are PBS at pH 7.4 including 5 µM GSH to mimic extracellular environment and PBS at pH 7.4 including 5 mM GSH to mimic intracellular environment, respectively. Peptide release were quantified by HPLC, and the dates were showed by Mean±SD.

To ascertain the cytomembrane penetrability of peptide-auric SNH, we prepared N-terminally fluorescein isothiocyanate (FITC)-labled ^FITC^PMI-Au SNH, ^FITC^BBI-Au SNH and ^FITC^DPA-Au SNH, and examined their cellular uptakes by flow cytometry. As shown in Figure 4A and Figure S5A-C, three peptide-auric SNH efficiently internalized into the HCT116 cells at the concentration of from 0.5 to 2 μM (based on quantification of thiol-cleaved peptide), whereas free ^FITC^PMI, ^FITC^BBI and ^FITC^DPA failed to traverse the cytomembrane at the concentration of 1 μM. To explore the cellular uptake pathway of Au SNH, 3 mM micropinocytosis inhibitor, Amiloride, were used to pre-incubate HCT116 cells. As expected, Amiloride inhibited ~60% cellular uptake of ^FITC^PMI-Au SNH (Figure S5D). Moreover, for further verification, 2 μM cytochalasin D were used to inhibit the actin function of cells. In line with the Amiloride, cytochalasin D also suppressed the cellular uptake of PMI-Au SNH (Figure S5D). These data demonstrated that PMI-Au can efficiently internalize into cells *via* actin-dependent micropinocytosis. After that, we explored the intracellular distribution of ^FITC^PMI-Au SNH by colocalization nanoparticle with early endosome, late endosome and lysosome. As shown in Figure S5E, hardly any PMI-Au SNH (green) colocalized with the red late endosomes or lysosomes, while partial of them overlap with early endosomes.

Therapeutic efficacy of therapeutic peptides targeting intracellular PPIs is bound up with the effective concentration of peptide cargo in cytosol, and thus, another necessary designed function of peptide-auric SNH is to contortedly release payloads inside the targeted cell. It has reported that the Au-S bond is a stable chemical connection in extracellular physiological conditions, but can be broken by a high concentration of thiols 46. Glutathione (GSH) is a common nonprotein thiol in organism, and found in millimole range inside the cell but micromole range outside the cell (Figure 4B) 33. To verify the stimuli responsive cargo release triggered by the differential concentration of GSH, analytical HPLC was used to monitor the release kinetics of PMI-Au SNP (Figure 4C), BBI-Au SNP (Figure 4D) and DPA-Au SNP (Figure 4E). In PBS buffer containing 5μM GSH at pH 7.4, all the three Peptide-Auric SNH generally maintain their integrality with <11% cargo release after a 12-h incubation (Figure 4C-E). In sharp contrast, adding GSH to 5 mM resulted in the disintegration of Au SNPs into small gold core (Figure S6) and subsequent ~90% cumulative release within another 6 h (Figure 4C-E), indicating a GSH-concentration-dependent cargo release. In short, these data validate our polymeric peptide-auric chemistry for the synthesis of SNH as a viable strategy for intracellular delivery and GSH-triggered release of therapeutic peptides.

### Peptide-auric SNH resurrected the anti-cancer activities of PMI, BBI and DPA* in vitro*

**Figure 5:**
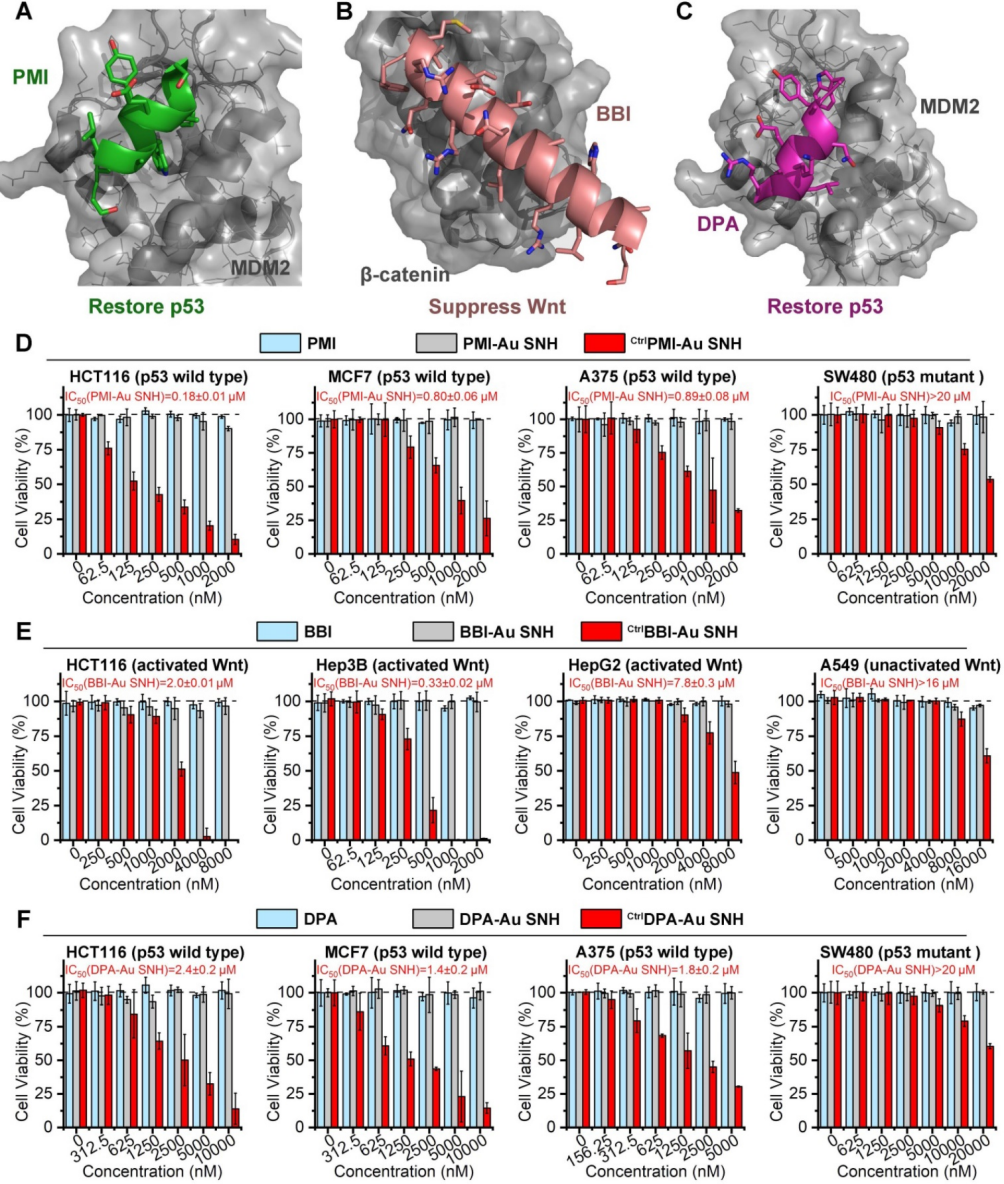
** Peptide-auric SNH resurrected the anti-cancer activities of PMI, BBI and DPA* in vitro*.** (**A-C**) Anti-cancer mechanisms of PMI (A) (PDB code:3LNZ), BBI (B) (PDB code: 3SL9) and DPA (C) (PDB code: 3IWY). (**D**) The cell viabilities in effect of the PMI-Au SNH, ^Ctrl^PMI-Au SNH (negative control) and PMI treatment measured by MTT. (**E**) The cell viabilities in effect of the BBI-Au SNH, ^Ctrl^BBI-Au SNH (negative control) and BBI treatment measured by MTT. (**F**) The cell viabilities in effect of the DPA-Au SNH, ^Ctrl^DPA-Au SNH (negative control) and DPA treatment measured by MTT. The dates were showed by Mean±SD.

As previous report, the dodecameric peptide PMI is designed for competitive antagonism MDM2 to restore p53, thereby reactivating the anti-cancer function of p53 to kill cancer cells (Figure 5A) 47. The 20-mer BBI was dedicated to targeting β-catenin toward disturbing the β-catenin/Bcl9 interaction and as a result of the blockage of the cancerogenic Wnt signaling pathway (Figure 5B) 48. Besides, dextrorotary peptide DPA has similar functions as PMI to restore p53 (Figure 5C) 45. Unfortunately, suffering from poor proteolytic stability and/or low membrane permeability, PMI, BBI and DPA fail to suppress cancer cells *in vitro*. To confirm that SNH can resurrect their anti-cancer activities, three negative (inactive) controls ^Ctrl^PMI-Au SNH, ^Ctrl^BBI-Au SNH and ^Ctrl^DPA-Au SNH were synthesized, where the two functionally most critical residues of PMI, BBI or DPA were mutant to Ala, respectively.

We firstly evaluated the *in vitro* anti-tumor activity of PMI, ^Ctrl^PMI-Au SNH and PMI-Au SNH against three cell lines carrying wild-type p53 and overexpressed MDM2: HCT116 (colon), A375 (melanoma) and MCF-7 (breast). PMI-Au SNH inhibited all three cell lines in dose-dependent manners, whereas^ Ctrl^PMI-Au SNH and PMI free were non-inhibitory (Figure 5D). Moreover, an obvious activity decline of PMI-Au SNH can be found in a colorectal cancer cell line harboring mutant p53, SW480 (Figure 5D). These results suggested that PMI-Au SNH inhibited cell viability of tumor cells in a fashion of p53 dependence, which was proved again by the up-regulation of p53 and p21 after PMI-Au SNH treatment (Figure S7). Next, the cytotoxicity of BBI, ^Ctrl^BBI-Au SNH and BBI-Au SNH were tested in three Wnt-hyperactivated cancer cell lines: HCT116 (colon), Hep3B (hepatoma) and HepG2 (hepatoma). As shown in Figure 5E, BBI-Au SNH dose-dependently inhibited cancer cell proliferation in contrast to the hardly any efficacy of BBI and ^Ctrl^BBI-Au SNH. Additionally, BBI-Au SNH also showed sub-efficacy to the Wnt-unactivated cell lins, A549 (lung cancer, Figure 5E), suggesting the Wnt dependent manner of BBI-Au SNH (Figure S7). As for DPA-Au SNP, similar results can be found in Figure 5F and Figure S7 as PMI-Au SNP. These results confirmed that peptide-auric SNH can resurrect the anti-cancer activities of PMI, BBI and DPA* in vitro*.

### *In vivo* biodistribution and antitumor activity of Pep-Au SNH

**Figure 6:**
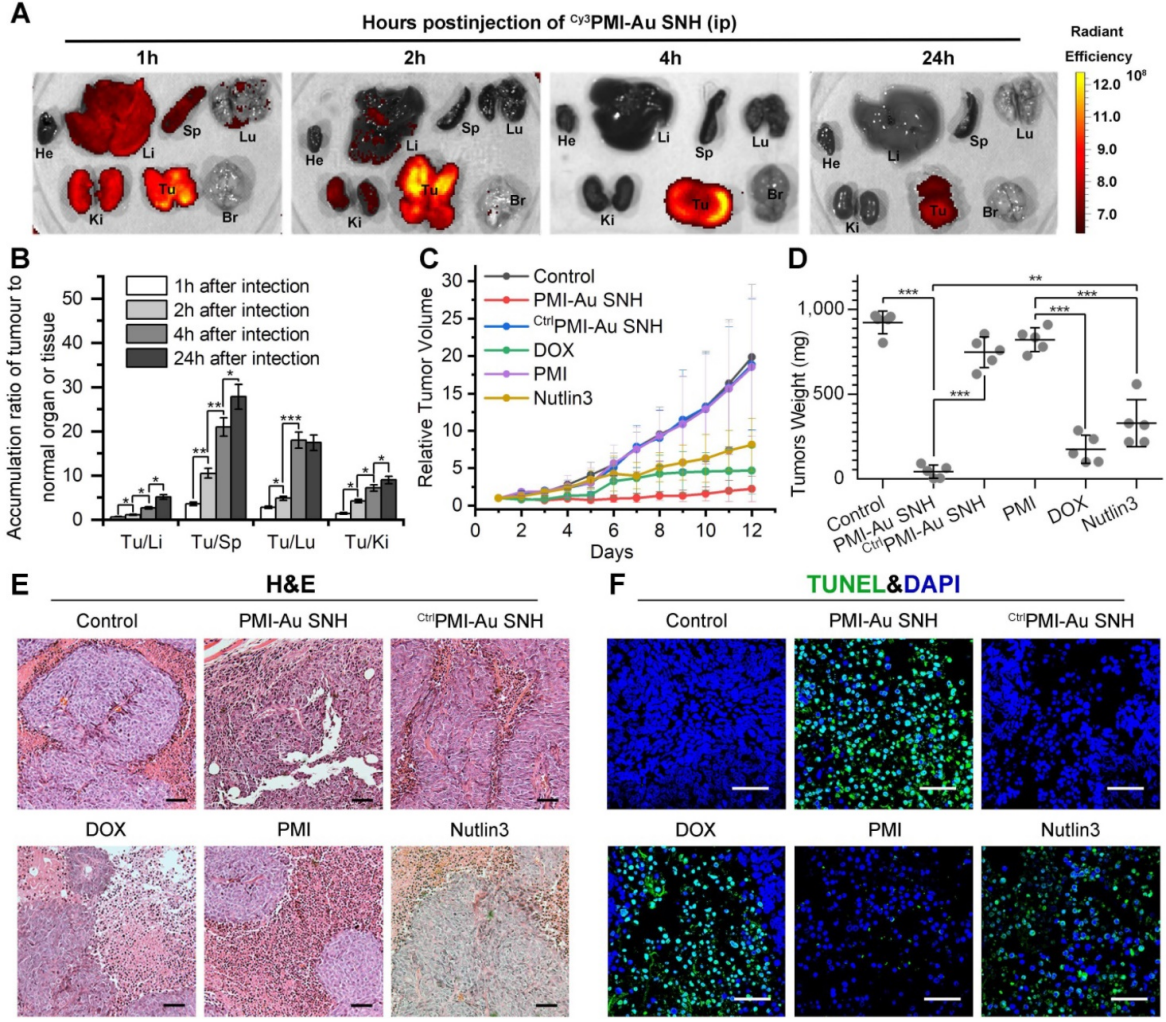
***In vivo* biodistribution and antitumor activity of PMI-Au SNH.** (**A**) Tissue distribution and safety of PMI-Au SNH. The fluorescence signal from the tumors and normal organ after PMI-Au SNH intraperitoneal injection (200 µL, 1mM Au) at 1, 2, 4 and 24 h. (**B**) Tumor-to-normal tissue ratios for PMI-Au SNH after intraperitoneal injection (200uL, 1mM Au) at 1, 2, 4 and 24 h. (n =3/group, mean ± s.d.). *p* values were calculated by t-test (*, p<0.05; **, p < 0.01; ***, p < 0.001). (**C**) Tumor growth curves in nude mice subcutaneously inoculated with 1×10^6^ HCT116 cells into the right flank. A statistical analysis was performed using a non-parametric Kruskal-Wallis test. Data are presented as mean ± s.e. (n =5). (**D**) Weights of the tumors excised at the end of the experiment. (**E**) H&E staining (×200) of HCT116 solid tumor tissues after 12-day treatments. (**F**) representative images of Tunel staining for tumor tissue taken by confocal laser scanning micrscope (CLSM) (scale bar: 60 µm).

To further challenge the function of Peptide-auric SNH, PMI that possesses all weaknesses of peptide therapeutic including poor proteolytic stability, low membrane permeability and hydrophobicity, was selected to evaluate its *in vivo* tumor accumulation and therapeutic efficacy after the nanoengineering. In solid tumors, the widespread incomplete blood vessel allowed nanoparticles ranging from 10 to 200 nm to leave the blood and enter into the ovarian malignancies 49, 50. Meanwhile, intratumoral underdeveloped lymphatic vessels restricted the particle exclusion, resulting in the tumor-specific spontaneous accumulation (passive targeting) of nanoparticle, and this series of phenomena is currently known as enhanced permeability and retention (EPR) effect 49, 51. We performed pharmacokinetics studies of PMI-Au SNH by examining its organ-specific distribution. To spectrophotometrically monitor PMI-Au SNH distribution in mice bearing subcutaneous xenografts of HCT116 tumors, we prepared ^Cy3^PMI-Au SNH, where Cy3 was C-terminally conjugated to the peptide, injected it intraperitoneally (200 μl injection containing 1 mM Au), and inspected the animals at 1, 2, 4 and 24 h using a quantitative *in vivo* optical imaging system. As shown in Figure 6A, accumulation of ^Cy3^PMI-Au SNH in the liver, spleen, lung and kidney quickly reached a maximum at 1 h post-injection, subsided thereafter, and was largely invisible within 4 hours. By contrast, a significantly high level of accumulation of ^Cy3^PMI-Au SNH in the tumor was maintained over time and declined only at the 24 h time point. Quantification of organ-specific and time-dependent differential accumulation indicated that ^Cy3^PMI-Au SNH preferentially accumulated in the tumor as opposed to the liver, spleen, kidney or lung (Figure 6B); the ratio of accumulation of ^Cy3^PMI-Au SNH in the tumor to other organs progressively increased over time (Figure 6B).

To evaluate the therapeutic efficacy of PMI-Au SNH *in vivo*, we used a xenograft tumor model in which HCT116 *p53*^+/+^ cells were subcutaneously inoculated to the flank of BALB/c nude mice. A 12-d treatment regimen was preformed involving intraperitoneal injections of PMI-Au SNH, doxorubicin (DOX), free PMI, ^Ctrl^PMI-Au SNH, Nutlin3 and saline, every other day, at a dose of 2.5 mg/kg. We used DOX (a first-line chemotherapy drug) and Nutlin (a small molecule antagonist of MDM2 52) as two positive control. As shown in Figure 6C, PMI-Au SNH was more active than DOX as well as Nutlin3 and potently inhibited tumor growth in experimental animals, while free PMI and ^Ctrl^PMI-Au SNH were, as expected, inactive; statistical analysis of the weights of tumors excised from mice sacrificed at the end of treatment confirmed this finding (Figure 6D).

**Figure 7:**
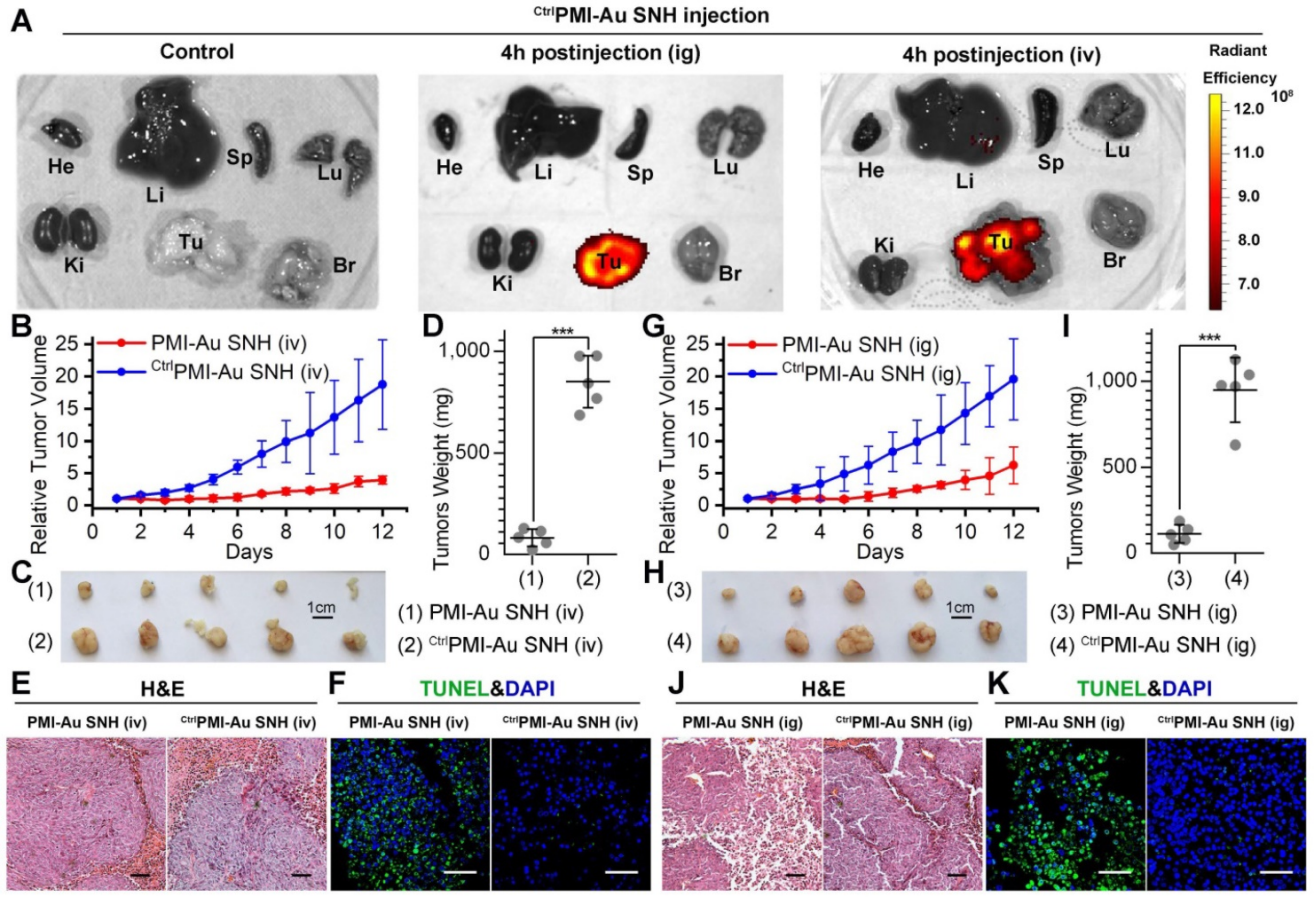
** PMI-Au SNH was efficacious by intravenous or intragastrical administration.** (**A**) Ex vivo fluorescent images of tumors and major organs from ^Cy3^PMI-Au SNH mice after 4h tail vein injection (iv) or oral gavage (ig). (**B**) Tumor growth curves in nude mice bearing HCT116 tumor with intravenous administration (n =5/group). (**C-D**) Photo (C) and weights (D) of the tumors excised at the end of the experiment. (**E**) H&E staining (×200) of HCT116 solid tumor tissues after 12-day treatments. (**F**) representative images of Tunel staining for tumor tissue taken by confocal laser scanning micrscope (CLSM) (scale bar: 60 µm). (**G**) Tumor growth curves in nude mice bearing HCT116 tumor with intragastrical administration (n =5/group). (**H, I**) Photo (C) and weights (D) of the tumors excised at the end of the experiment. (**J**) H&E staining (×200) of HCT116 solid tumor tissues after 12-day treatments. (**K**) representative images of Tunel staining for tumor tissue taken by confocal laser scanning micrscope (CLSM) (scale bar: 60 µm).

To further characterize the *in vivo* antitumor activity of PMI-Au SNH at the histopathological level, we analyzed tumor tissues using hematoxylin and eosin (H&E) staining and terminal deoxynucleotidyl transferase-mediated dUTP nick end labeling (TUNEL) techniques. As expected, PMI-Au SNH treatment significantly increased levels of apoptosis, contrasting the PMI and ^Ctrl^PMI-Au SNH treatment groups (Figure 6E-F). Collectively, PMI-Au SNH was significantly more active than Nutlin3 and DOX in tumor suppression *in vivo*. Notably, PMI-Au SNH was also efficacious when administered at the same dose intravenously (Figure 7A-F). As shown in Figure 7A, intravenous PMI-Au SNH tumor-specifically accumulated. Additionally, intravenous PMI-Au SNH significantly suppressed the tumor growth (Figure 7B-D), which can be further proved by the increased apoptotic cell in PMI-Au SNH-treated group (Figure 7E-F). Collectively, these results showcased a versatile treatment adaptable to tumors in different locations.

To further explore the long-term cancer proliferation inhibition effect, BBI-Au SNH was used to intraperitoneally inject into the BALB/c nude mice bearing Hep3B xenograft tumor for a 21-day treatment. BBI-Au SNH was administrated every other day at a dose of 2.5 mg/kg. Expectedly, the tumor inhibitory rate in BBI-Au SNH-treated mice reached ~80% (Figure S8A-B) at day 21, while BBI-Au SNH significantly inhibited the Wnt signaling pathway (Figure S8C-E). Of note, BBI-Au SNH showed an outstanding therapeutic safety that proved by the steady blood biochemical indexes (Figure S8M-F).

### PMI-Au SNH was efficacious by intragastrical administration

Although some nanomedicines are being tested in clinical trials or have been approved 22, 53, few of them is currently amenable to parenteral methods 54. As for cancer therapy, oral delivery allows a long-time continuous administration, which has been proved safer and more effective than the current intermittent therapy by injection or infusion 55, 56. Suffering from the poor stability and the mucosal permeability, nanoparticles always tend to degradation prior to absorption in gastrointestinal tract. Fortunately, our SNH possess a stable spherical structure with diameter of 30 nm, which is favorable to pass the gastrointestinal barriers through paracellular passage between intestinal epithelial cells 55. To further confirm the stability of PMI-Au SNH under the stomach's acidic environment, the colloidal stability and proteolytic resistance of PMI-Au SNH were examined at pH 4.0. As expected, PMI-Au SNH kept its monodispersity over 48 h at the pH 4.0 (Figure S9A), and more than 75% peptide maintained integrity against chymotrypsin at pH 4.0 (Figure S9B). To verify the gastrointestinal accumulation and permeability of PMI-Au SNH, we resorted to a sensitive technique, inductively coupled plasma mass spectrometry (ICP-MS), for the detection and quantitation of 197Au in the colon, stomach and blood. Time-dependent ICP-MS measurements of 197Au were expressed as Injected Dose percent per Gram/Milliliter of Tissue/Blood (ID%/g) in Figure S9C-E. Quantification of organ-specific differential accumulation showed that PMI-Au SNH has ability to accumulate at colon and stomach, and pass through the gastrointestinal barrier into the blood after gastric perfusion administration. To further confirm it, 200 μl ^Cy3^PMI-Au SNH were poured into the stomach in mice bearing subcutaneous xenografts of HCT116 tumors. After 4 hours, bright fluorescence can be found just in tumor (Figure 7A), indicating that intragastrical administration is compatible to the tumor-specific accumulation of PMI-Au SNH.

Next, mice bearing HCT116 tumors were used again to investigate therapeutic efficacy of intragastrically administered PMI-Au SNH. During the 12-day treatment regimen, intragastrical PMI-Au SNH inhibited tumor growth by 72.6% (Figure 7G). At the end of the experiments, all tumors were collected and weighed (Figure 7H-I). Statistic data of tumor weights in Figure 5D supported the findings again in Figure 7G. Moreover, PMI-Au SNH treatment significantly increased levels of apoptosis, contrasting the ^Ctrl^PMI-Au SNH treatment groups (Figure 7J-K). Collectively, PMI-Au SNH was also efficacious by intragastrical administration, demonstrating that Peptide-Auric SNH is suitable for oral use.

### PMI-Au SNH restored p53 activity *in vivo*

**Figure 8:**
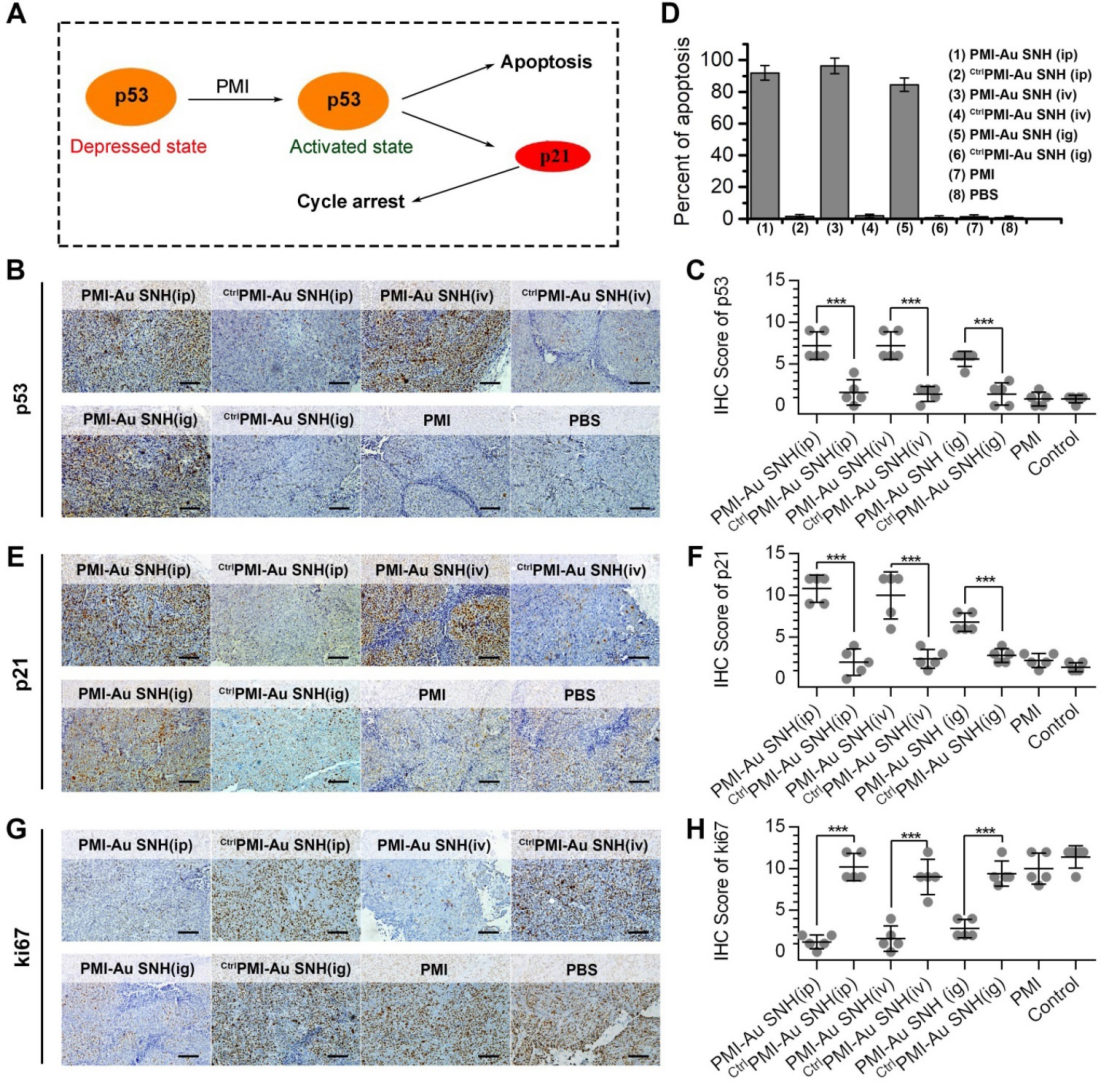
** The molecular mechanism of PMI-Au SNH in tumor suppression.** (**A**) schematic diagram of tumor therapeutic of the function of PMI in restoration of p53 activity. (**B and C**) representative IHC staining (B) and IHC score (C) for p53 protein in tumor tissues from mice with the indicated treatments (scale bar: 50 µm). (**D**) TUNEL score of tumor tissues from mice with the indicated treatments. (**E and F**) representative IHC staining (E) and IHC score (F) for p21 protein in tumor tissues from mice with the indicated treatments (scale bar: 50 µm). (**G and H**) representative IHC staining (G) and IHC score (H) for ki67 protein in tumor tissues from mice with the indicated treatments (scale bar: 50 µm). *p* values were calculated by t-test (*, p <0.05; **, p <0.01; ***, p <0.001).

The p53 induces potent growth inhibitory *via* p21 and apoptotic responses *via* BH3 protein and, playing a pivotal role in preventing tumorigenesis (Figure 8A) 57, 58. Over one-third of all human malignancies involve the function inhibition of p53 by MDM2 and/or MDMX 59, 60. To explore the mechanism of PMI-Au SNH in tumor suppression, all tumors were harvested on day 12. Semi-quantitative immunohistochemistry analysis of p53 unveiled a significant increase in the levels of both p53 in tumor tissues from mice treated with PMI-Au SNH, but not PMI and ^Ctrl^PMI-Au SNH (Figure 8B-C). As a result, upregulated p53 induced the apoptosis of tumor cells (Figure 8D). Moreover, consistent with this finding, PMI-Au SNH treatment significantly increased the level of p21 (Figure 8E-F), which further induced the cycle arrest that were accompanied by the decreased ki67 (Figure 8G-H).

### *In vivo* safety evaluation of PMI-Au SNH

To minimize the drug toxicity, a two-stage targeting strategy was adopted in polymeric Au-peptide nanoparticle. As mentioned above, aided by EPR effect (first targeting), PMI-Au SNH can concentrate to the tumor and retain at least 24 hours (Figure 6A). In addition, therapeutic peptide PMI specifically target MDM2, who just overexpressed in cancer cells, further guaranteeing the safety of PMI-Au SNH.

**Figure 9:**
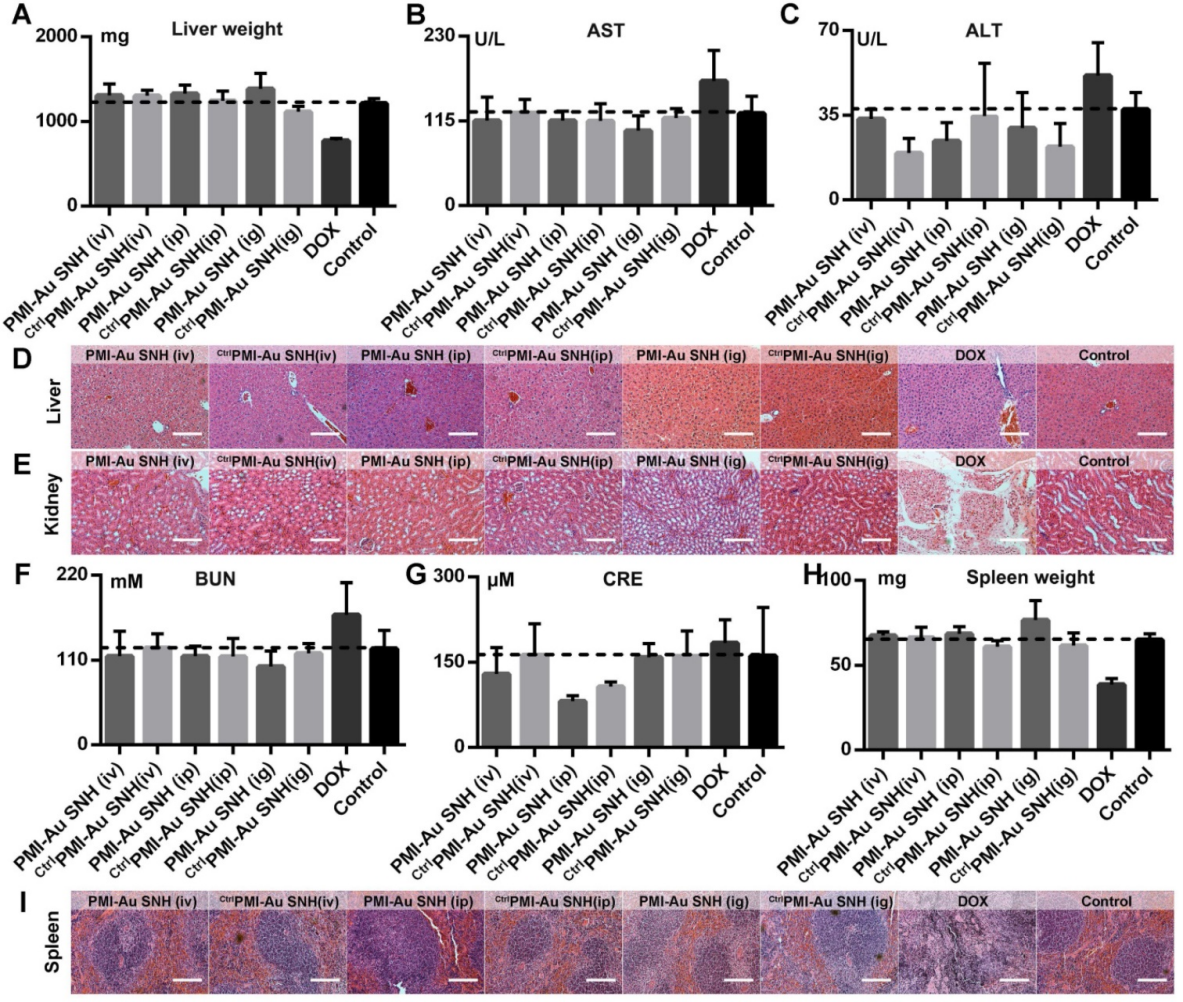
***In vivo* safety evaluation of PMI-Au SNH.** (**A**) liver weight of mice after the 12-day treatments. (**B and C**) the activities of aspartate aminotransferase (AST (B)) and alanine transaminase (ALT (**C**)) related to liver function in mice after the indicated treatments. (**D** and** E**) the representative histological H&E staining images of liver (D) and kidney (E) in mice after the indicated treatments (scale bar: 50µm). (**F-I**) measurement of renal function indicators in mice after the indicated treatments. (BUN (F), blood urea nitrogen; CRE (G), serum creatinine). (**H**) Spleen weight of mice with the indicated treatments. (**I**) the representative histological H&E staining images of spleen in mice (scale bar: 50 µm).

To evaluate the toxicity of PMI-Au SNH including the nephrotoxicity *in vivo*, we carried out a comprehensive toxicity study using the subcutaneous xenograft model of HCT116 tumors from efficacy studies. PMI-Au SNH was intraperitoneally, intravenously or intragastrically injected, every other day for a duration of 12 days, at a dose of 2.5 mg/kg, with saline, ^ctrl^PMI-Au SNH and DOX as controls. Little difference in body weight between the ^ctrl^PMI-Au SNH - and mock-treated groups was observed (Figure S10), indicating a lack of acute toxicity of ^ctrl^PMI-Au SNH to the animals. As expected for chemo drugs, DOX precipitously caused a significant loss of body weight in the DOX-treated group. By contrast, mice in the PMI-Au SNH-treated groups, regardless of the route of drug administration, all gained weight over time due, presumably, to the suppression of tumor growth and lack of toxic effects of PMI-Au SNH *in vivo*. The safety of both PMI-Au SNH and ^ctrl^PMI-Au SNH and toxicity of DOX were further confirmed by changes, or lack thereof, in the function of the liver and kidney (Figure 9A-G), in the weight and H&E staining of spleen (Figure 9H-I) and in the number of white blood cells and thrombocytes (Figure S11). Moreover, heart function (Figure S12), the level of red blood cells (Figure S13) and the histological H&E staining of lung (Figure S14) supported the above findings and the conclusion that PMI-Au SNH is sufficiently safe with a significant therapeutic potential.

## Discussion

Undoubtedly, nanoengineering therapeutic peptides into stable nanostructures will not only greatly extend the scope of nanotechnology, but notably, it is also meaningful for the development of peptide drug to overcome its pharmaceutical obstacles. Up to know, the rational design of ideally bioactive, stable and bioavailable peptide-derived therapeutics remains an extremely difficult task. Although various methods have been developed to improve the cytomembrane penetrability and tumor targeting of peptides, mainly through the chemical modification of peptide 2, and covalently or non-covalently carrying peptide by delivery vehicles 5. However, most of these widely used peptide modifications in the first strategy, such as backbone circulation, stapling and non-natural amino acids incorporation, often have relatively complex components and largely depend on time-consuming and expensive organic syntheses 61, 62. The latter based mainly on multifarious drug delivery system, including, but not limited to, liposomes, micelles, and nanoparticles, which always suffer from rapid remove by the liver and spleen, non-specific cellular uptake, and unstable nanostructure 63, 64. Therefore, there is a critical need for alternative strategies towards the clinical application of therapeutic peptides, and our sample methods for nanoengineering peptides was born out of this expectation.

Moreover, more and more gold nanoparticles (AuNP)-conjugated peptide therapeutics have been developed to deliver drugs and biomolecules and applied for clinical trials owing to its intrinsic advantage including essential inertion, low-toxicity and economic costs 22, 26, 27. Yet the complex chemical properties (hydrophobicity, charge and redox) of peptide are always adverse to the steady state of colloidal AuNP after conjugation, resulting in the subsequent aggregation or even precipitation under the physiological condition of the elevated ionic concentration 29, 30. Meanwhile, the weakened colloidal stability often accompanies premature release of therapeutic peptide and enhanced reticuloendothelial system uptakes, and ultimately leads to off-target toxicity and therapeutics failing 26, 31. Fortunately, our general-purpose nanohybrid fabricated by polymeric Au(I)-peptide precursor completely overcome pharmaceutical obstacles for peptide-gold derived nanomedicine.

Controllable and predictable fabrication of bioactive peptide-derived nanomaterials hasn't been fully implemented yet, because the current supramolecular chemistry cannot control inter-or intramolecular interactions of peptide accurately 12, 65. In fact, most of the peptide-based nanostructures are designed and fabricated *via* one or several non-covalent bonds including ionic, hydrophobic, hydrogen bonding, and π-π stacking 11, 19, and thus unpredictable forces from the complex primary/secondary peptide structures would make the stability, repeatability and function of the nanostructures uncertain. Additionally, a batch of self-assembled peptide nanomaterials have shown a variety of biomedical applications such as biosensors, biomineralization and drug delivery, nearly all these self-assemblies were based on some specific peptide sequences, which possess special physical and chemical properties tending to form supramolecular structure 11. Therefore, this general strategy to nanoengineer peptides into a predictable nanostructure filled these gaps and successfully overcame pharmaceutical obstacles of therapeutic peptides, particularly those targeting PPIs.

## Conclusions

The data presented here provide compelling evidence that the design of peptide-auric SNH is a general and viable class of peptide nano-engineering strategy to transform intracellular-PPI-targeting peptides into a potential drug. Overcoming pharmacological deficiencies, SNH successfully rescued the anti-cancer activity of PMI, BBI and DPA that, on their own, failed to kill cancer cells. More importantly, peptide-Auric SNH showed the favorable tumor-specific accumulation and potent efficacy *in vivo* when administered intraperitoneally, intravenously or intragastrically, showcasing a versatile treatment adaptable to tumors. With superior therapeutic safety, this *de nove* peptide nano-engineering strategy has shown great potential in clinical translation. In short, our work may supply a new feasible strategy to bridge the distance between peptide discovery and clinical application, and accelerate the conversion of intracellular PPI targets to therapeutics.

## Supplementary Material

Supplementary materials and methods, figures.
