# A general-purpose Nanohybrid fabricated by Polymeric Au(I)-peptide precursor to wake the function of Peptide Therapeutics: Erratum

**DOI:** 10.7150/thno.73289

**Published:** 2022-04-26

**Authors:** Jin Yan, Fanpu Ji, Siqi Yan, Weiming You, Fang Ma, Fanni Li, Yinong Huang, Wenjia Liu, Wangxiao He

**Affiliations:** 1 National & Local Joint Engineering Research Center of Biodiagnosis and Biotherapy, The Second Affiliated Hospital of Xi'an Jiaotong University, Xi'an, 710004, PR. China.; 2 Department of Tumor and Immunology in precision medical institute, Western China Science and Technology Innovation Port, Xi'an, 710004, PR. China.; 3 Department of Infectious Diseases, The Second Affiliated Hospital of Xi'an Jiaotong University, Xi'an, 710004, PR. China.; 4 Key Laboratory of Environment and Genes Related to Diseases, Xi'an Jiaotong University, Ministry of Education of China, Xi'an, China; 5 Department of Talent Highland, The First Affiliated Hospital of Xi'an Jiaotong University, Xi'an 710061, PR. China.; 6 Shaanxi Institute of Pediatric Diseases, Xi'an Children's Hospital, Xi'an, Shaanxi 710003, PR. China.; 7 The Second Affiliated Hospital of Xi'an Jiaotong University, Xi'an, 710004, PR. China.

Authors regret that the original version of our paper unfortunately contained some incorrect representative images in Figure 6. The H&E image of Nutlin3 in Figure 6E and the TUNEL image of PMI in Figure 6F had been misused during figure assembly. The correct version of the Figure 6E and 6F appears below.

Authors confirm that the corrections made in this erratum do not affect the original conclusions. We apologize for any inconvenience that the errors may have caused.

## Figures and Tables

**Figure 6:**
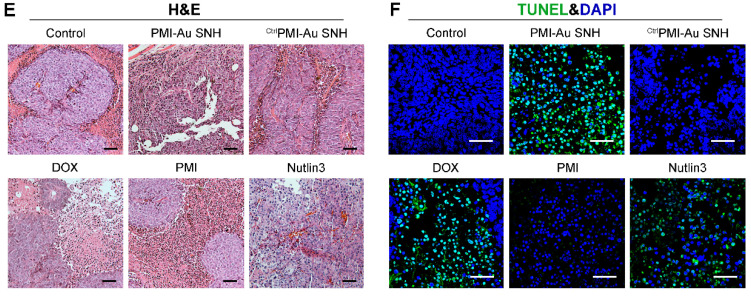
***In vivo* biodistribution and antitumor activity of PMI-Au SNH.** (E) H&E staining (×200) of HCT116 solid tumor tissues after 12-day treatments. (F) representative images of Tunel staining for tumor tissue taken by confocal laser scanning micrscope (CLSM) (scale bar: 60 μm).

