# Supplementary material for: A general-purpose Nanohybrid fabricated by Polymeric Au(I)-peptide precursor to wake the function of Peptide Therapeutics
Source: Theranostics. 2020 Jul 9;10(19):8513–27. doi: 10.7150/thno.47243 (PMC7392018; doi:10.7150/thno.47243)
Supplement: Supplementary file 1 — Supplementary materials and methods, figures. [file thnov10p8513s1.pdf]

Supplementary materials

A general-purpose nanohybrid fabricated by polymeric Au(I)-peptide precursor to wake the function of peptide therapeutics

Jin Yan[1,2,†], Fanpu Ji[1,3,4,†], Siqi Yan[5,†], Weiming You[1,2], Fang Ma[1,2], Fanni Li[5], Yinong Huang[6,*], Wenjia Liu[1,2,*] Wangxiao He[5,7*]

[1]National & Local Joint Engineering Research Center of Biodiagnosis and Biotherapy, The Second Affiliated Hospital of Xi'an Jiaotong University, Xi'an, 710004, PR. China.

[2]Department of Tumor and Immunology in precision medical institute, Western China Science and Technology Innovation Port, Xi'an, 710004, PR. China.

[3]Department of Infectious Diseases, The Second Affiliated Hospital of Xi'an Jiaotong University, Xi'an, 710004, PR. China.

[4]Key Laboratory of Environment and Genes Related to Diseases, Xi'an Jiaotong University, Ministry of Education of China, Xi'an, China

[5]Department of Talent Highland, The First Affiliated Hospital of Xi'an Jiaotong University, Xi'an 710061, PR. China.

[6]Shaanxi Institute of Pediatric Diseases, Xi'an Children's Hospital, Xi'an, Shaanxi 710003, PR. China.

[7]The Second Affiliated Hospital of Xi'an Jiaotong University, Xi'an, 710004, PR. China.

*Corresponding author:

Email: ynhuang@xjtu.edu.cn (Y. Huang)

Email: wenjia@xiterm.com (W. Liu)

Email: hewangxiao5366@xjtu.edu.cn (W. He)

†These authors contributed equally to this work.

Materials and methods

General remarks

$HAuCl_4·XH_2O$ was purchased from Aladdin Chemicals. All synthetic peptide sources were obtained from CS Bio (Shanghai) Ltd. All other chemicals used in this study were purchased from Sigma-Aldrich unless otherwise specified. Acetonitrile and water (HPLC grade) were purchased from Fisher Scientific Ltd. All products were used as received without further purification.

Synthesis of peptide (PMI, BBI and DPA)

All peptides were synthesized on appropriate resins on an CS bio 336X automated peptide synthesizer using the optimized HBTU activation/DIEA *in situ* neutralization protocol developed by an HBTU/HOBT protocol for Fmoc-chemistry SPPS. After cleavage and deprotection in a reagent cocktail containing 88% TFA, 5% phenol, 5% H2O and 2%TIPS, crude products were precipitated with cold ether and purified to homogeneity by preparative C18 reversed-phase HPLC. The molecular masses were ascertained by electrospray ionization mass spectrometry (ESI-MS).

Fabrication of PMI-Au SNH, BBI-Au SNH and DPA-Au SNH

An aqueous solution of tetrachloroauric acid (HAuCl4·XH2O, 1 mL, 10 mM) was mixed with 9 mL 50 mM HEPES buffer (pH 7.4, 50 mM). After 10min magnetic stirring, the solution color changed from golden yellow to wine red, which is the Au core. Meanwhile, add 1ml tetrachloroauric acid (10mM) solution into 9 mL peptide buffer (50 mM HEPES, 20% ethyl alcohol) dissolved 5mg peptides (PMI-Cys, BBI-Cys or DPA-Cys). After then, mix the 10ml

peptide-Au with the 10ml Au-core, following by a 10-min magnetic stirring. Finally, removed the excess reactants by dialysis tubing (cutoff, 10 KDa) and washed twice by distilled water.

Fabrication of PMI-AuNPs, BBI-AuNPs and DPA-AuNPs

An aqueous solution of tetrachloroauric acid ($HAuCl4·XH2O$, 1 mL, 10 mM) was mixed with 9 mL 50 mM HEPES buffer (pH 6.0, 50 mM). After 10min magnetic stirring, the solution color changed from golden yellow to wine red, which is the Au core. Meanwhile, add 1ml peptide aqueous solution (including 1mg PMI-Cys, BBI-Cys or DPA-Cys), following by a 10-min magnetic stirring. Finally, removed the excess reactants by dialysis tubing (cutoff, 10 KDa) and washed twice by distilled water.

Physicochemical properties of PMI-Au SNH and its intergradations

The morphology and lattice structure were observed on transmission electron microscopy (TEM), which was performed on an HT7700 operated at an acceleration voltage of 100 kV. The hydrodynamic size distribution (1 mg/mL in PBS, 1 mL) was obtained from the dynamic light scattering (DLS) measurement (Malvern Zetasizer Nano ZS system). For Zeta potential measurement, the nanoparticles (1 mg/mL, 1 mL) were incubated with PBS at different pH at 37 ºC for 30 min, and measured by dynamic light scattering (DLS). The surface chemical structure of modified nanocrystals was evaluated by Fourier transform infrared (FT-IR) spectroscopy (Nicolet 6700) and UV−vis absorption spectra (Shimadzu 3000 spectrophotometer).

Quantification of drug loading and GSH-responded drug release

To quantify drug loading Pep-Au-SNH were dissolved in PBS buffer (pH 7.4) containing 6 M guanidine hydrochloride and 1 M dithiothreitol to break the conjunction between peptides and gold atoms, and the amounts of released peptides were then quantified by high-performance liquid chromatography (HPLC). To test GSH-responded drug release, AuNp-DPAs were dissolved in PBS buffer (pH 7.4) containing 10 mM glutathione (GSH), and the nanoparticles were then removed by 14000 g centrifuge. Following this, the supernatants were quantified by HPLC and authenticated by ESI-MASS. Separations were performed at a flow rate of 1 mL/min with a gradient from 5 to 65% of B in 30 min (eluent A: 0.1% TFA/H2O, eluent B: 0.1% TFA in CH_3CN).

Cell culture and viability anslysis

Human colon cancer cell lines HCT116 $p53^{+/+}$ carrying wild-type p53 and the isogenic HCT116 $p53^{-/-}$ (p53 deletion) were kindly provided by Prof. Bert Vogelstein (Ludwig Center at John Hopkins, Baltimore, USA), and maintained in McCoy's 5A medium with 10% FBS. Human hepatoma cell line Hep3B, human lung adenocarcinoma cell line A549, human breast cancer cell line MCF-7 and human malignant melanoma cell line A375 were also purchased by ATCC and maintained in DMEM with 10% FBS. All cells were maintained at 37°C in an atmosphere of 5% CO2. The *in vitro* cytotoxicity was measured using a standard MTT (Thermo Fisher scientific) assay in the above cell lines. Cells were plated in 96-well plates at a density of 2500 cells/well (100 uL). After 24 h, cells were treated with prescribed samples at the indicated concentrations and times, respectively. All cells were maintained at 37°C in an atmosphere of 5% CO_2.

Cellular uptake of PMI-Au SNH

Cellular uptake of PMI-Au SNH was detected using flow cytometer (BD Biosciences, NJ). FITC was firstly labeled to the N-terminal of PMI. Similarly, ^{FITC}Pep-Au SNH was prepared as above. First, ^{FITC}Pep-Au SNH and ^{FITC}Pep were re-dispersed in culture medium at a concentration of 2μM, and HCT116 cells were cultured in corresponding growth medium for 24 h. The medium was then replaced with the conditional medium containing ^{FITC}PMI-Au SNH and ^{FITC}PMI at pH 7.4, and further cultured at 37°C for 6 h. Flow cytometry analysis were then carried out after washing the cells with PBS twice to remove the excess ^{FITC}PMI-Au SNH or ^{FITC}PMI.

***In vivo* bio-distribution analysis**

HCT116 cells (4×10^6 cells/site) were implanted subcutaneously into hip of four- to five-weeks-old male athymic nude mice. Four weeks after cell inoculation, tumor-bearing mice were injected with 200 μL Cy3-labelled ^{FITC}PMI-Au SNH. IVIS Spectrum *In Vivo* Imaging System was then used to take the *ex vivo* images of mice. Light with a wavelength at 490 nm was used as the excitation source. In addition, mice were humanely killed at predetermined times, and the heart, liver, spleen, lung, kidney and tumor were immediately collected from each mouse. The fluorescence intensity in all organs was further analyzed by the IVIS Spectrum *In Vivo* Imaging System.

Animal studies

All animal experiments were performed by following Institution Guidelines and were approved by the Laboratory Animal Center of Xi'an Jiaotong University. HCT116 cells were harvested by incubation with 0.05% trypsin-EDTA when they reached near confluence. Cells were

then resuspended in sterile PBS. HCT116 cells (4 × 10^6 cells/site) were implanted subcutaneously into hip of four- to five-weeks-old male athymic nude mice. When the tumors reached average volume of ~ 50 mm^3, the mice were randomly divided into different groups (five mice per group). Tumor length and width were measured with calipers, and tumor volume was calculated using the following equation: tumor volume (V) = length × $width^2/2$. Before H&E or immunohistochemical staining, the tumor, liver, kidney, heart, spleen and lung tissues were fixed with formaldehyde, dehydrated, and sliced into 5.0 μm sections. In addition, blood routine examination, the function of liver, kidney and spleen, and some biochemical enzyme indexes were carried out at the Clinical Laboratory of the First Affiliated Hospital of Xi'an Jiaotong University according to the standard clinical laboratory procedures.

Immunohistochemical (IHC) staining

Sections were cut at 5 μm thickness, deparaffinized and rehydrated. Endogenous peroxidase activity was blocked with hydrogen peroxide/methanol, and antigen retrieval was performed in a pH 9.0 TE (Tris-EDTA) buffer by autoclave for 10 min. The resultant tissue sections were then incubated with primary antibodies against p53, p21 and Ki67 at 4°C overnight. After incubation with labeled streptavidin-biotin (LSAB) complex for 15 min, the slides were stained and visualized by using the iView DAB detection system (ZSGB-BIO, P.R. China). Each stained section was evaluated by a minimum of 10 randomly selected ×20 high-power fields for further statistical analysis.

To evaluate immunostaining intensity (I), we used a numeric score ranging from 0 to 3, reflecting the intensity as follows: 0, no staining; 1, weak staining; 2, moderate staining; and 3,

intense staining. To evaluate immunostaining area (A), we used a numeric score ranging from 1 to 4, reflecting the intensity as follows: 1, positive area <10%; 2, 10%<positive area <50%; 3, 50%<positive area <90%; and 4, positive area>90%. Using an Excel spreadsheet, the mean score was obtained by multiplying the intensity score (I) by the percentage of positive area and the results were added together (total score: I×A).

Toxicity studies

To assess potential toxicities of repeatedly infusing PMI-Au SNH, we monitored body weight of all mice over the course of treatment and measured hematological indexes as well as organ function indexes after 13-day treatment. Control mice were only implanted with xenograft tumor, but did not receive any treatment. Forty-eight hours after the final infusion, mice were anesthetized, and blood was collected for complete blood count (CBC) determinations, including a white blood cell (WBC) count with differential, a red blood cell (RBC) count, haemoglobin and a platelet count. Besides, blood serum was collected, and alanine aminotransferase (ALT), aspartate transaminase (AST), blood urea nitrogen (UN) and creatinine (CRE) were measured by using quantitative enzyme-linked immunosorbent assay (ELISA) kits according to the manufacturer’s instructions. Animals were then euthanized with carbon dioxide to retrieve organs, which were washed with deionized water before fixation in 4% paraformaldehyde. The tissues were processed routinely, and sections were stained with haematoxilin and eosin (H&E).

Statistical analysis.

Statistical analyses were performed using two-sided Student's *t*-test or ANOVA. *P* <0.05

was considered significant. Data were expressed as mean ± s.d. or s.e.

Supplementary Figures

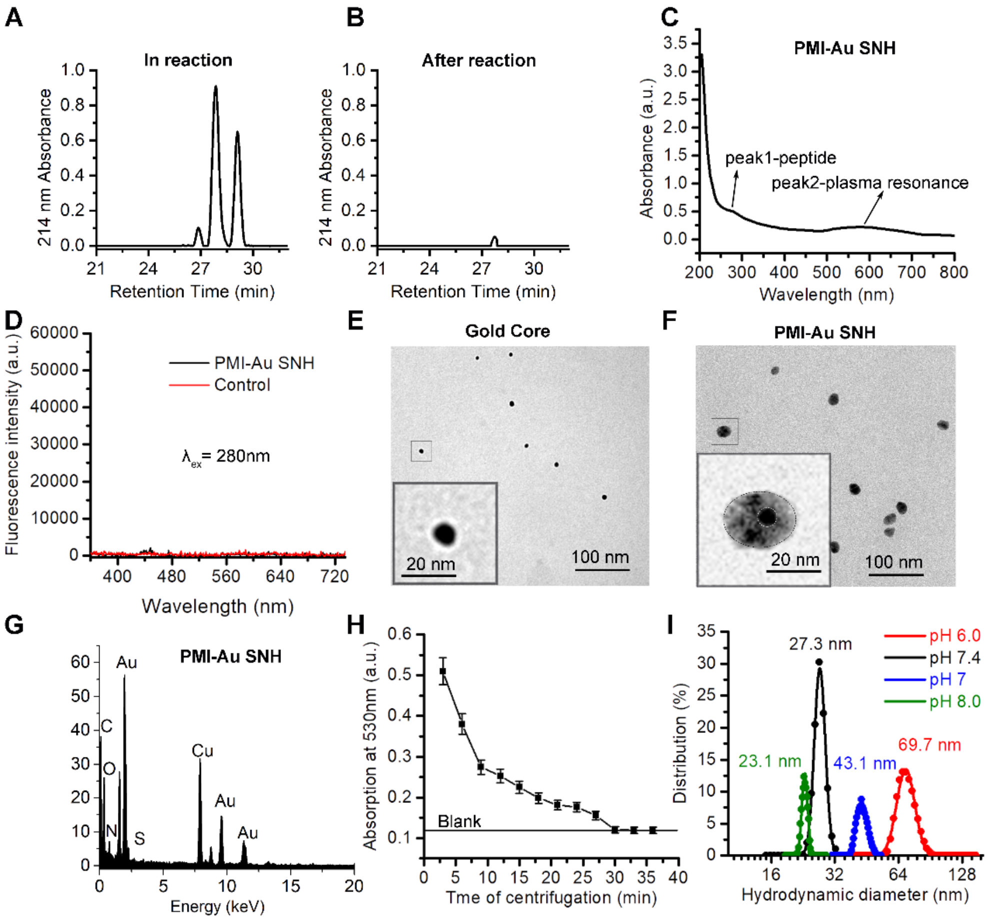

Figure S1. (A&B) Monitoring chemical reaction I by HPLC. After the reaction, the peaks of PMI-SH, PMI-S-S-PMI and PMI-S-Au disappeared. (C) UV–Vis absorption spectra of PMI-Au SNH. (D) The Emission fluorescence spectrum of PMI-Au SNH. (E) High-resolution TEM (HRTEM) image of Gold Core. (F) High-resolution TEM (HRTEM) image of PMI-Au SNH. (G) EDS analysis of PMI-Au SNH. The signals of Cu in panel (G) originated from the carbon-coated copper grid. (H) Time-dependent absorbance detection at 530nm of the supernatant after 10000g centrifugation of PMI-Au SNH solution. (I) Hydrodynamic diameter distributions of PMI-Au SNH synthesized in pH 6.0, 7.0, 7.4 and 8.0.

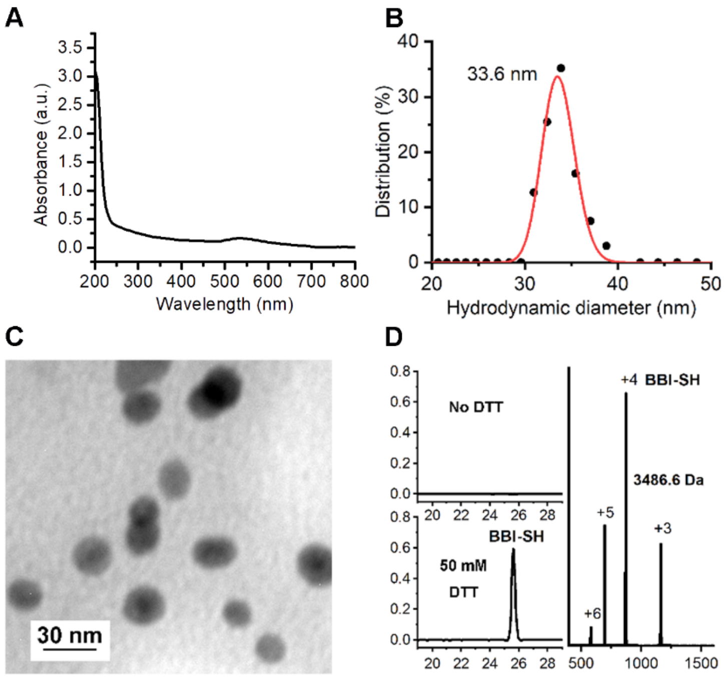

Figure S2. Characterization of BBI-Au SNH. (A) UV–Vis absorption spectra of BBI-Au SNH. (B) Hydrodynamic diameter distributions and the solution photo of BBI-Au SNH. (C) Transmission electron micrograph images (TEM) of BBI-Au SNH. (D) HPLC analysis of the residual BBI-SH in the liquid supernatant after the BBI-Au SNH synthesis and centrifugation (top left). The bottom left panel is the HPLC analysis of BBI-Au SNH-redissolved solution including 50 mM dithiothreitol (DTT), and the right is the ESI-MASS result of the peak in the bottom left panel.

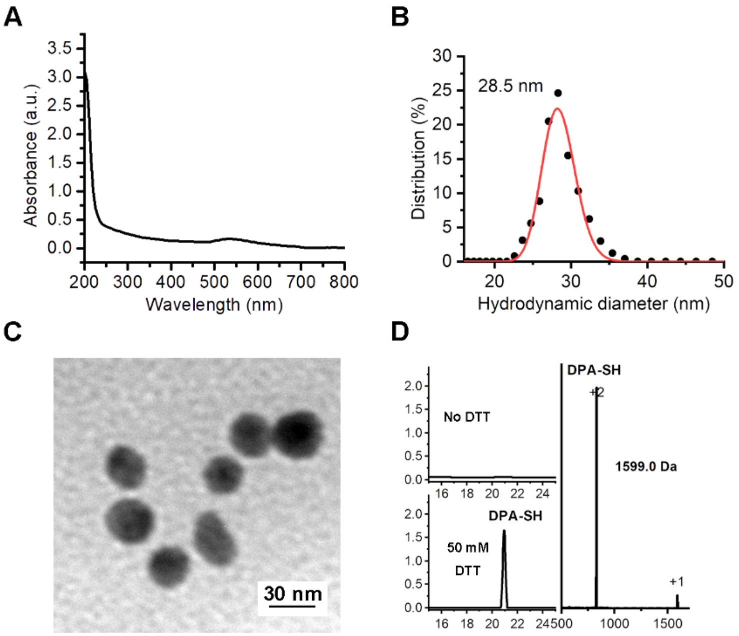

Figure S3. Characterization of DPA-Au SNH. (A) UV–Vis absorption spectra of DPA-Au SNH. (B) Hydrodynamic diameter distributions and the solution photo of DPA-Au SNH. (C) Transmission electron micrograph images (TEM) of DPA-Au SNH. (D) HPLC analysis of the residual DPA-SH in the liquid supernatant after the DPA-Au SNH synthesis and centrifugation (top left). The bottom left panel is the HPLC analysis of DPA-Au SNH-redissolved solution including 50 mM dithiothreitol (DTT), and the right is the ESI-MASS result of the peak in the bottom left panel.

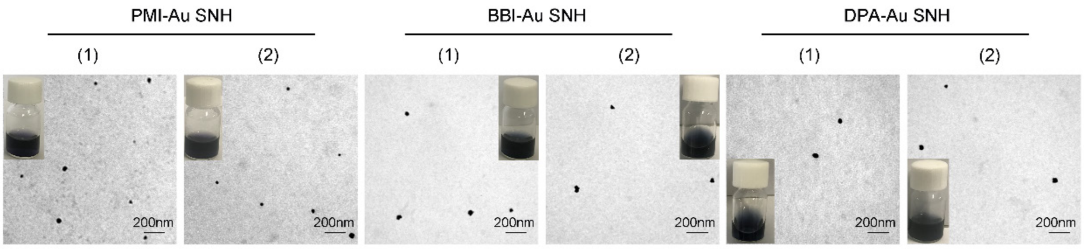

Figure S4. Optical photographs and TEM images of three Au SNH after (1) 12h incubation in PBS at pH 7.4 or (2) 12h incubation in PBS containing 20%FBs and 5μM GSH at pH 7.4.

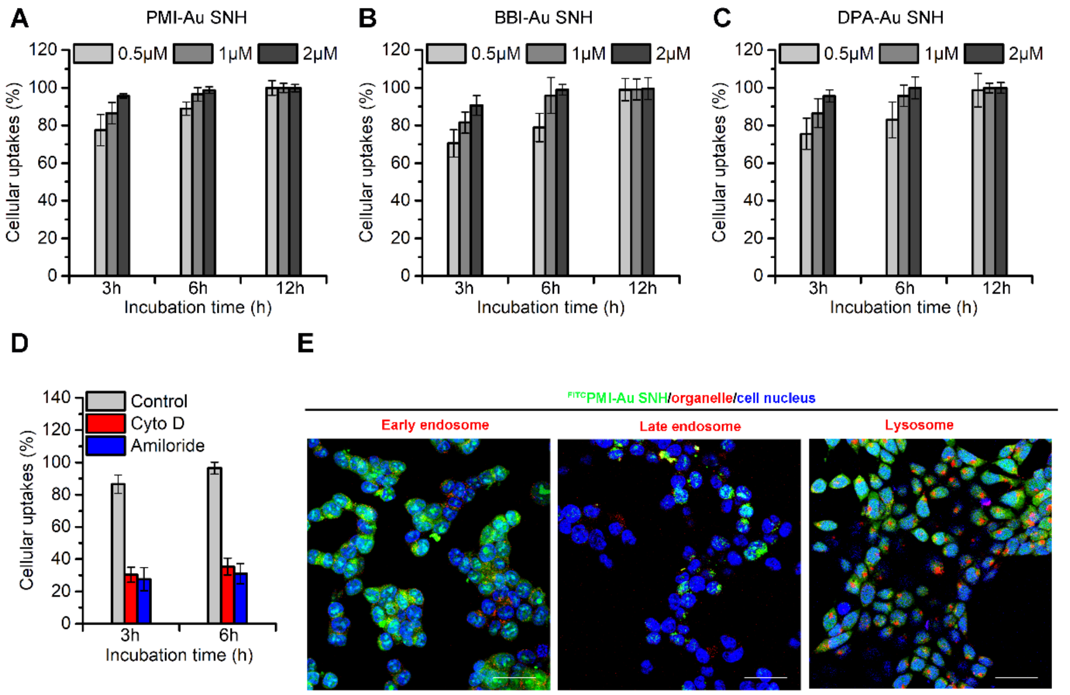

Figure S5. (A-C) flow cytometry analysis of cell uptakes of 0.5, 1 and 2 μM ^{FITC}PMI–Au SNH (A), ^{FITC}BBI–Au SNH (B), ^{FITC}DPA–Au SNH (C) into HCT116 cancer cells after 6h incubations. (D) Cellular uptake of 1μM ^{FITC}PMI–Au SNH with Amiloride or Cyto D preincubation into HCT116 cells. (E) Co-localization of FITC-labeled PMI–Au SNH with lysosome and early and late endosomes. The subcellular organelles are marked in red. (scale bar: 60 μm).

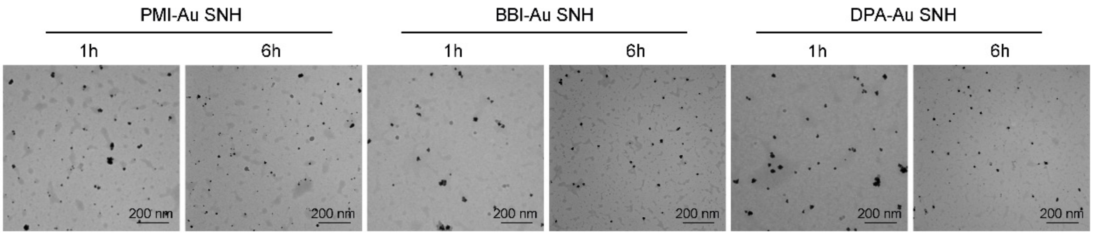

Figure S6. TEM images of three Au SNH after 1 h or 6 h incubation in PBS at pH 7.4 including 5 mM GSH.

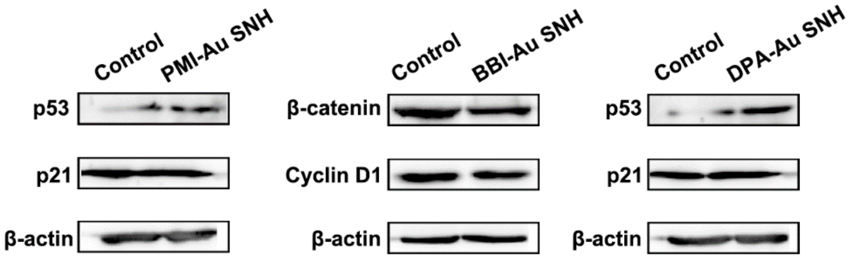

Figure S7. HCT116 were treated with 1 μM PMI-Au SNH, BBI-Au SNH and DPA-Au SNH for 48 h, and western blot was performed to analyze the expressions of p53,p21, β-catenin and Cyclin D proteins. β-actine was used as loading control.

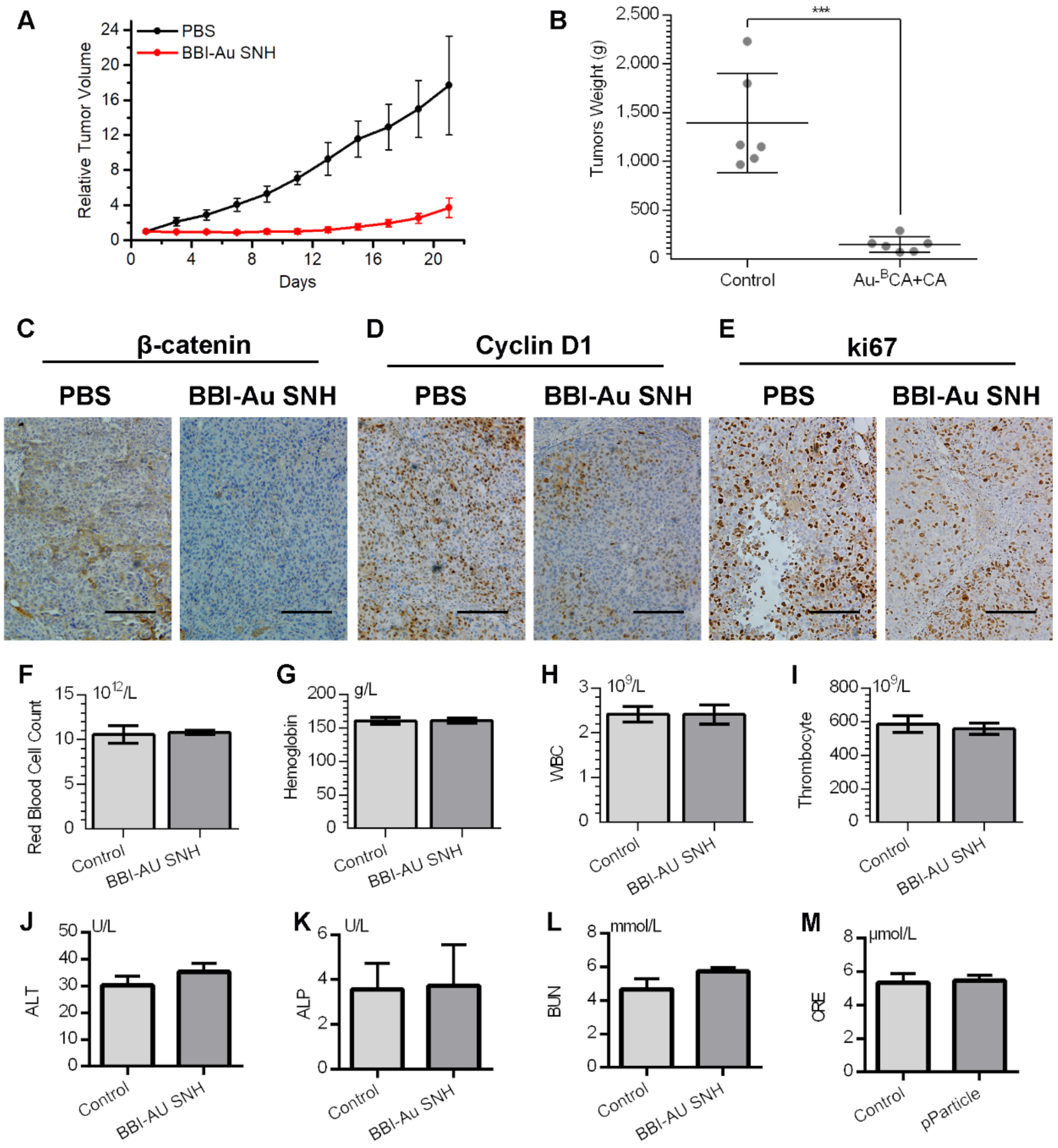

Figure S8. *In vivo* antitumor activity of BBI-Au SNH. (A) Tumor growth curves in nude mice subcutaneously inoculated with 1×10^6 HEP3B cells into the right flank. A statistical analysis was performed using a non-parametric Kruskal-Wallis test. Data are presented as mean ± s.e. (n =6).

(D) Weights of the tumors excised at the end of the experiment. (C-E) Representative images of β -catenin, Cyclin D1 and ki67 staining for tumor tissue. (F-M) Blood biochemical indexes after 21-day treatment.

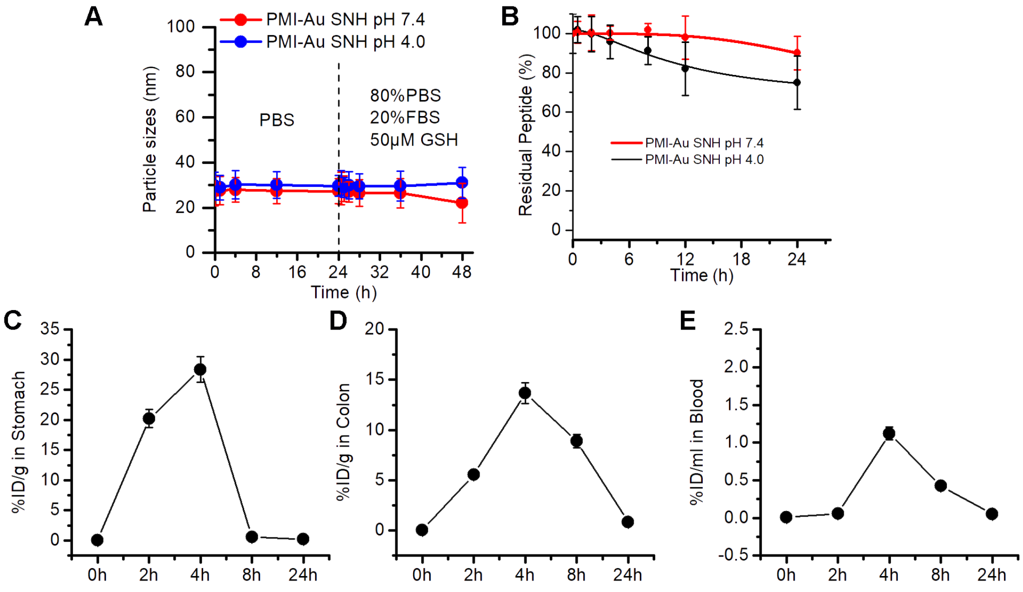

Figure S9. (A) The dimensional change of PMI-Au SNH with time in PBS or PBS including 20% FBS and 50μM GSH at pH 7.4 or 4.0 to simulate extracellular physiological environment. (B) Proteolysis resistance of Pep-Au SNH under PBS at pH 7.4 or 4.0 containing 10 mM oxidized glutathione, 10% serum, and 0.5 mg/ml chymotrypsin. (C) ICP-MS quantification of gold content (from PMI-Au SNH) in stomach, colon and blood collected from health c57/b6 mice. The data were mean ± SD.

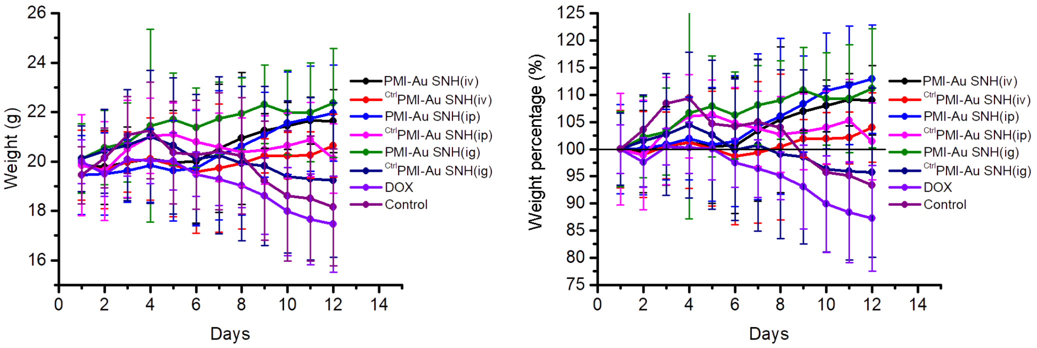

Figure S10. Weight of mice (left panel) and relative weight (right panel) of each group with the indicated treatments. The data were presented as mean ± s.d. of values from 6 mice in each group.

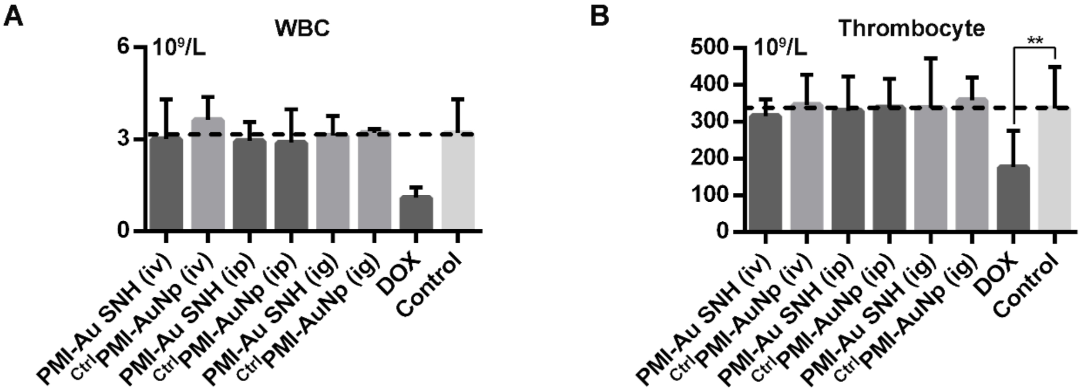

Figure S11. Blood cells analysis after indicated treatments. A&B, the count of white blood cells (WBC, A) and thrombocyte(B) in mice with the indicated treatments. p values were calculated by t-test (unmarked p >0.05 and ** p<0.01).

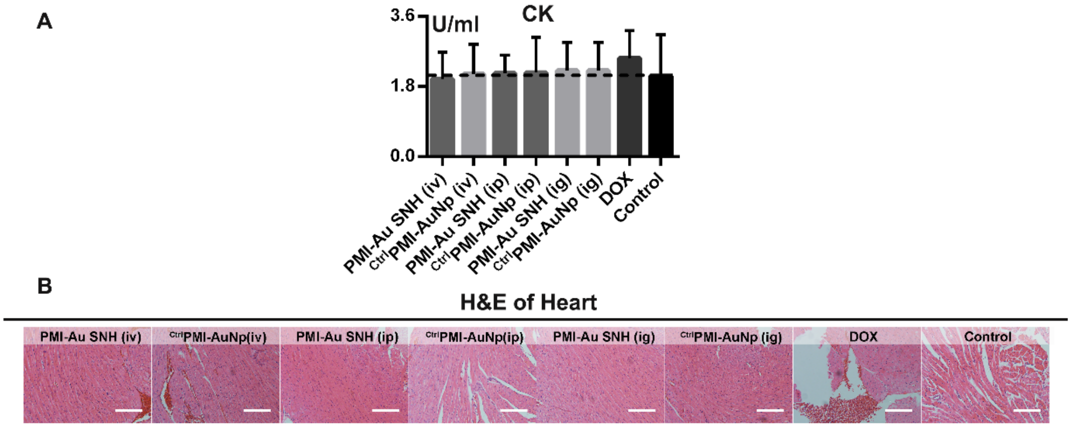

Figure S12. Toxicity evaluation of the heart in mice with different treatments. (A) The activity of serum creatine kinase (CK) in mice with the indicated treatments. Clinically, CK is assayed in blood tests as a marker of damage of CK-rich tissues such as in myocardial infarction (heart attack). The data were presented as mean ± s.d. of values from 6 mice in each group. *p* values were calculated by t-test (unmarked $p > 0.05$). (B) The representative H&E staining of heart sections in mice with the indicated treatments (scale bar: 50 μm).

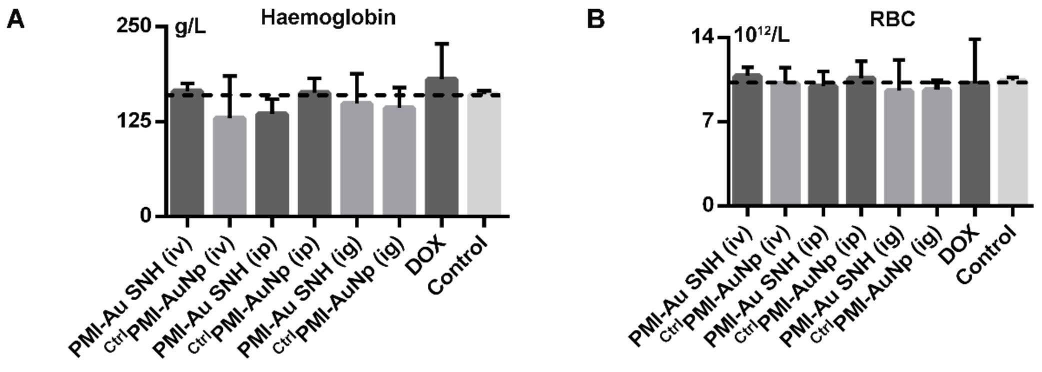

Figure S13. The count of hemoglobin (A) and red blood cells (RBC, B) in mice with the indicated treatments. The data were presented as mean ± s.d. of values from 6 mice in each group. *p* values were calculated by t-test (unmarked $p > 0.05$).

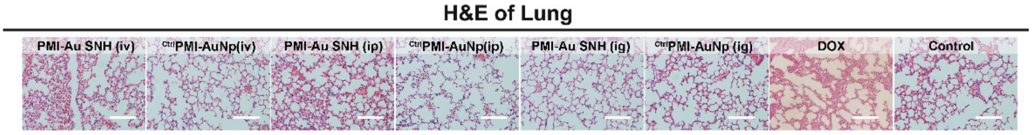

Figure S14. Toxicity evaluation of the lung in mice with different treatments. The representative H&E staining of lung tissues in mice with the indicated treatments (scale bar: 50 μm).
